# Developing an Evaluation System for Quality of Health Educational Short Videos on Social Media (LassVQ) Using Nominal Group Technique and Analytic Hierarchy Process: Qualitative Study

**DOI:** 10.2196/72661

**Published:** 2025-09-18

**Authors:** Yang Hu, Yiran Yang, Wei Li, Yan Zhou, Jing Sun

**Affiliations:** 1 Peking University Third Hospital, Beijing, China; 2 School of Nursing, Peking University, 38 Xueyuan Rd, Haidian District, Beijing, China, 86 13520238688; 3 HSSE Technical Support Center, China National Oil and Gas Exploration and Development Company Ltd, Beijing, China; 4 Clinical Laboratory, Shanxi Bethune Hospital, Shanxi Academy of Medical Sciences, Tongji Shanxi Hospital, Third Hospital of Shanxi Medical University, Taiyuan, China; 5 Tongji Hospital, Tongji Medical College, Huazhong University of Science and Technology, Wuhan, China

**Keywords:** Lasswell’s 5W communication model, LassVQ, health educational short videos, HESV, social media, video quality

## Abstract

**Background:**

With the increasing use of social media platforms for health communication, the quality of health educational short videos (HESVs) has become a key concern. However, no standardized framework exists to evaluate the quality of health videos on social media, highlighting the need for a comprehensive evaluation system.

**Objective:**

This study aimed to develop a valid and structured evaluation tool for assessing the quality of HESVs on social media.

**Methods:**

The initial evaluation indicators obtained from the literature review and brainstorming undertaken in the study group were provided to the nominal group reference Lasswell’s 5W communication model, and 2 rounds of nominal group technique (NGT) were carried out to screen, add, revise, and adjust indicators, and reach a consensus of evaluation system. The indicators were then ranked based on their significance, as scored by the experts using the analytic hierarchy process. The content validity was assessed by experts who rated the relevance of each indicator on a 4-point Likert scale.

**Results:**

The primary indicators include communicator, communication content, communication channel, and communication effect, along with 13 secondary indicators and 34 tertiary indicators. In total, 11 experts were enrolled in the NGT, 45% (5/11) of experts had a doctoral degree, and 80% (9/11) of them were ranked as an associate professor or professor. The average values of the expert judgment coefficient and authority coefficient were 0.93 (SD 0.08) and 0.85 (SD 0.10), respectively. In round 1 of NGT, the “communication target” of 5 primary indicators, 7 of 20 secondary indicators, and 66 of 94 tertiary indicators did not reach a consensus, and therefore, they were not deleted and proceeded to the next round of NGT. In round 2 of NGT, 1 primary indicator, 7 secondary indicators, and 59 tertiary indicators were deleted based on the consensus criteria. Among primary indicators, communication content was found to be the most influential, accounting for 45.68%. Among secondary indicators, credibility, scientificity, availability, and social attention were the most influential indicators, with priorities of 56.67%, 24.26%, 74.62%, and 39.89% in their respective categories. Among tertiary indicators, “become a hot search recommended by the platform” was the most influential indicator with a weight of 0.07. The content validity of all the evaluation indicators was 0.73-1.0, and the scale-level content validity index (average) was 0.87 (SD 0.15), which was indicated as acceptable.

**Conclusions:**

The evaluation system for the quality of HESVs on social media (LassVQ; the Lasswell’s Video Quality scale) was developed, and its validity was acceptable. The proposed evaluation system can be used in conjunction with qualitative methods to gain a holistic perspective on the multidimensional quality of HESVs on social media.

## Introduction

Social media provides useful forums for health care providers and consumers to exchange health information; the boom of short video platforms also offers new potential for health-related information dissemination [1,2]. A total of 83% of American adults [3] and 74.2% of the total Chinese population [4] are active social media users. Furthermore, 87.6% of YouTube users watched health-related content, with 84.7% noted some influence on their health decisions [5]. On social media channels, posts with videos had 3 times greater chance of receiving likes, almost 4 times greater chance of receiving comments, and 2.5 times greater chance of being shared [6]. Social media–based interventions have been associated with reduction in weight, BMI, and waist circumference by meta-analyses [7]. Around 50% of people act on physical activity and diet videos shared on social media during the COVID-19 lockdown [8].

Videos can capture visual information, facilitate self-paced learning, and engage learners, making them a valuable tool for learning procedures and complex skills that may be difficult to acquire through reading or description alone [9]. Social media permits video submissions from all users; it can be subjective or inaccurate, which dilutes its overall quality [2]. In total, 30% to 87% of health information on social media is false, misleading, or causing harm [10]. False news spreads 3 to 20 times faster and deeper than the truth on social media [11]. Repeated exposure to such news tends to increase agreement [12], which is linked to potential harmful health decisions, poor medical compliance, discontinuation of medication, heightened anxiety, and so on [13].

A potential solution would be to use a standardized rating system to evaluate the quality of posted videos and display the score on the page alongside the videos [14]. In a review of 2113 videos, fewer than 1 in 10 of the studies used an externally validated quality scoring tool to evaluate the quality of medical educational videos, while the majority relied on tools or limited questions developed by authors [14]. The currently most commonly used tools are the DISCERN instrument [15], *Journal of the American Medical Association* (*JAMA*) benchmarks [16], and the Health on the Net principles [17]. These tools were originally developed for written health materials or websites in the 1990s, which may limit their suitability for assessing health educational short videos (HESVs) on social media [18]. HESVs require high-quality images and visuals, well-written scripts, and clear sound with minimal background noise; however, these features are not currently covered by existing evaluation tools [15,17-19].

In dynamic social media environments, both video content and engagement metrics are supposed to be in the HESVs quality assessment [20]. The quality of social media HESVs encompasses several dimensions, including content accuracy, reliability, understandability, interactivity, technical quality, and others [21-23]. A comprehensive and targeted quality evaluation system may contribute to the effective assessment of health educational short videos on social media, which remains an area of ongoing concern. Lasswell’s 5W communication model (the 5W model) is considered a foundational communication model and has been applied in certain new media contexts [24,25]. The analytic hierarchy process (AHP) is a widely used multiattribute weighting method for addressing complex decision-making problems in health care [26]. This study aims to develop a comprehensive evaluation system (LassVQ: the Lasswell’s Video Quality scale) for HESVs on social media, with preliminary evidence of validity, to facilitate more structured evaluation and understanding of video quality.

## Methods

### Overview

This methodological, multiphase study comprises 5 main stages to develop the LassVQ scale for assessing the HESVs on social media. This study was reported in accordance with COSMIN (Consensus-Based Standards for the Selection of Health Status Measurement Instruments) reporting guideline [27]. A completed COSMIN checklist is provided in Checklist 1.

### Ethical Considerations

This study was approved by the Biomedical Ethics Review Board of Peking University (IRB00001052-24115) and complied with the Declaration of Helsinki principles. All participants provided informed consent. Participants were assured that their privacy and confidentiality would be strictly protected, and all data were anonymized before analysis. No personal identifying information was collected or stored. No compensation was provided to participants.

### Stage 1: Generation of the Initial Evaluation Indicators

To develop an initial item pool of evaluation indicators, a literature review was conducted by predefined search terms, including “video,” “social media,” “social networking,” YouTube, TikTok, Douyin, quality, reliab*, understandability, actionab*, credib*, interactiv*, comprehensive*, accuracy, completeness, and clarity. Systematic searches were carried out across 5 major databases—PubMed (n=1864), Web of Science (n=6191), Embase (n=2817), CNKI (n=3543), and WANFANG (n=411) databases, spanning from the inception of each database to May 2024. Eligible records were limited to those published in English or Chinese and included peer-reviewed journal articles, academic reports, and conference proceedings relevant to the development or application of evaluation indicators. After removing duplicates (n=5401), 9425 records were screened based on titles and abstracts. Records deemed irrelevant or inaccessible, or that did not meet the inclusion criteria (n=9119), were excluded. The full texts of the remaining 306 articles were reviewed in detail, resulting in 72 studies meeting the final inclusion criteria. The research was conducted by 2 researchers (YH, YY) who participated in the literature search and screening. The entire research process was supervised and guided by one professor (JS). The professor was engaged in health education research for many years. The complete list of included studies is provided in Appendix S1 in Multimedia Appendix 1.

The study group summarized relevant research evidence, including preliminary research of literature review, brainstorming undertaken in the study group, literature reviews of the tools to assess printed health education materials. Then, a structured brainstorming session was subsequently organized with the study group, which included both senior experts and younger technical professionals. During the meeting, participants reviewed the draft framework and items, suggested additional items, identified potential overlaps, and proposed modifications. All inputs were recorded in real time by a designated notetaker (YY) to ensure completeness and accuracy. The resulting item pool integrated evidence from the literature (as the foundation) and expert opinions (to contextualize and refine items). Based on the 5W model, the initial version of the evaluation system comprised 5 primary indicators and a total of 119 indicators.

### Stage 2: Development of Consensus on Indicators

#### Composition of the Nominal Group

In May 2024, a sample of experts with experience in different aspects of health education was identified for the nominal group [28]. To be eligible to participate in the nominal group, participants needed to have intermediate or above professional titles, published works in relevant professional fields, and more than 8 years of experience in the current field. No conflicts of interest among participants were noted. In total, 13 people were invited, and 11 agreed to participate in the study. The 2 who declined to take part did so due to time conflicts between their clinical work and meeting schedules. The 11 participants comprised 4 health education research facilitators, 1 health education researcher, 2 HESV practitioners, 3 clinical health education practitioners, and 1 library information science expert.

#### Generation of the Nominal Group Questionnaire

Potential outcome domains and definitions are to be measured. In total, 5 primary indicators, 20 secondary indicators, and 94 tertiary indicators were listed, informed by Lasswell’s communication framework, evidence synthesis, and relevant assessment skills.

Potential methods of assessment are used to measure the domains and items. The necessity of each domain and item was rated using a 9-point Likert scale (1‐3=not essential, 4‐6=neutral, 7‐9=essential) [28]. Necessity was defined as “how necessary is it that this domain of measurement is included in the future assessment of the quality of HESVs on social media.”

The basis for measurement and scoring formed the references while measuring. One is the titles and sources of key references labeled after the indicators. Furthermore, 2 important resources, the printed full text of the original literature of the assessment tools, included the tools themselves.

#### Postal Invitation of the Nominal Group Experts

A letter of invitation that includes meeting time and place, process, and participants was mailed to the remaining experts of the meeting 2 weeks in advance of the consensus meeting. The precoded questionnaire and evidence synthesis were sent to the experts one week before the meeting.

#### Nominal Group Consensus Meeting

The 1-day NGT meeting was set up in a conference room on May 17, 2024, with a “U”-shaped table and a wide-screen monitor, including 4 sections. First, an introductory lecture was given to describe the meeting’s goals, the formation of the group, and the NGT process, particularly what experts need to do. Second, the precoded group rating questionnaire and evidence synthesis were presented. Participants had around 15 minutes to complete their individual ratings. Participants were asked to explore core domains, rewrite and add potential domains and their associated definitions, investigate reasons for any differences in ratings, and consider candidate outcome measures. Completed questionnaires were extracted by the conference secretary (Xiaojing Zhong). Data statistics and analysis were performed by 2 trained research assistants (YY, Ziling Pang). Third, a semistructured group discussion was completed after the first round of individual rating. Participants were asked to make suggestions. As some were more vocal than others, facilitators asked each participant to speak in turn, in the order in which they were sitting, clockwise. To ensure that everyone has the opportunity to speak independently, the seating order is ranked from lowest to highest in terms of authority. Fourth, items that were not agreed upon (deleted or retained) were regenerated for the round 2 questionnaires.

#### Data Analysis

The distribution of ratings for each domain and outcome measure was summarized, including the frequency, mean, SD, and IQR. The level of consensus reached by the group was set at 80% [29]. Indicators that reached group consensus with strong support, 80% of the group rated the indicator between 7 and 9. The NGT method specifies agreement as 7 out of 9 members’ panel rating within a specific 3-point range. With 11 participants, consensus was established at 9 out of 11 participants, or 80% [29].

The expert judgment coefficient (Ca) for each item was the sum score of each expert’s judgment basis for each item, representing the experts’ judgment. The impact degree scores of each judgment basis are illustrated in Appendix S2 in Multimedia Appendix 1. The familiarity coefficient (Cs) represents the familiarity of the expert, and the authority coefficient (Cr; Cr=[Ca+Cs]÷2), with the results expressed as mean (SD). If Cr was above 0.7, it was generally considered a relatively high degree of an expert authority. The degree of coordination of expert opinions is Kendall’s coordination coefficient W. Kendall’s coordination coefficient W (statistical significance set at *P*<.05) was used to assess whether the experts’ scoring of each item was consistent. The larger the value of the coordination coefficient W, the higher the degree of expert coordination.

### Stage 3: Empowerment of Evaluation Indicators

#### Overview

We used the AHP to analyze and determine the weight of each indicator [30]. Each respondent compares the relative importance of each pair of items using a specially designed questionnaire [31].

#### Data Analysis

This study uses Yaahp software (MetaDecision Software Technology Co, Ltd) to calculate the weights and verify the consistency of AHP. Yaahp is a kind of visual modeling and calculation software. According to the importance value assigned to each factor, we determined the Saaty scale, established the hierarchical model, constructed the judgment matrix, and conducted a hierarchical ranking and consistency test. The Saaty’s 9-point intensity of relative importance weight scale is illustrated in Appendix S3 in Multimedia Appendix 1. The consistency test was done by calculating the consistency index (CI) and the consistency ratio (CR). The consistency index is defined by the equation: $CI=\gamma_{max}-n/n-1$, CR=CI/RI, where $\gamma_{max}$ is the largest eigenvalue of a preference matrix, and n is the number of parameters. Random index (RI) values have been tabulated as a function of n, illustrated in Appendix S4 in Multimedia Appendix 1. Consistency ratios higher than 0.1 suggest untrustworthy judgments, indicating that the comparisons and scores should be revised. When the AHP model is run through the consistency check, the weight of all factors at each level and ranking can be calculated. The combined weight of the 3-level items is multiplied by 100, and the integer value is taken according to the rounding principle.

### Stage 4: Content Validity Test of the Evaluation System

#### Overview

The panel of experts assessed the relevance of the LassVQ evaluation system, and items were rated on a 4-point Likert scale, where 1 indicated “not relevant,” 2 indicated “marginally relevant,” 3 indicated “quite relevant,” and 4 indicated “strongly relevant.”

#### Data Analysis

The I-CVI (Item-level Content Validity Index) was calculated from the expert’s relevance rating for items 3 or 4, whereas the S-CVI/Ave (Scale-level Content Validity Index, Average) was derived by averaging the proportional relevance judgments of all experts. The number of experts in the relevance agreement was divided by the total number of experts. The interrater agreement among the experts was determined using the modified kappa statistic. The probability of chance agreement (Pc) for each item was determined using the formula: $Pc=\left[\left(N!/A!\right)\left(N-A\right)!\right]*0.5^{N}$. *N* is the number of experts, and *A* is the number of experts who agree that the item was relevant [32]. Kappa was determined using the following formula: *k*=(item-level content validity index*−Pc*)/(1−Pc). The kappa value above 0.74 was considered excellent, 0.60 to 0.74 as good, and 0.54 to 0.59 as fair [32].

### Stage 5: Pilot Testing

A pilot study with 15 participants (9 viewers and 6 producers of HESVs) was conducted to evaluate readability and comprehensibility of the draft scale, following the NGT. The main study did not include these participants. Data were collected through face-to-face interactions to ensure detailed feedback on the scale items during the pilot phase. Participants rated each item using a 5-point Likert scale (1=very poor, 5=excellent) for both readability and comprehensibility. Open-ended feedback was also solicited to identify potential ambiguities. The subsequent survey was conducted with the main participants after adjustments were made based on feedback received during the pilot test.

## Results

### Overview

**Figure 1.**
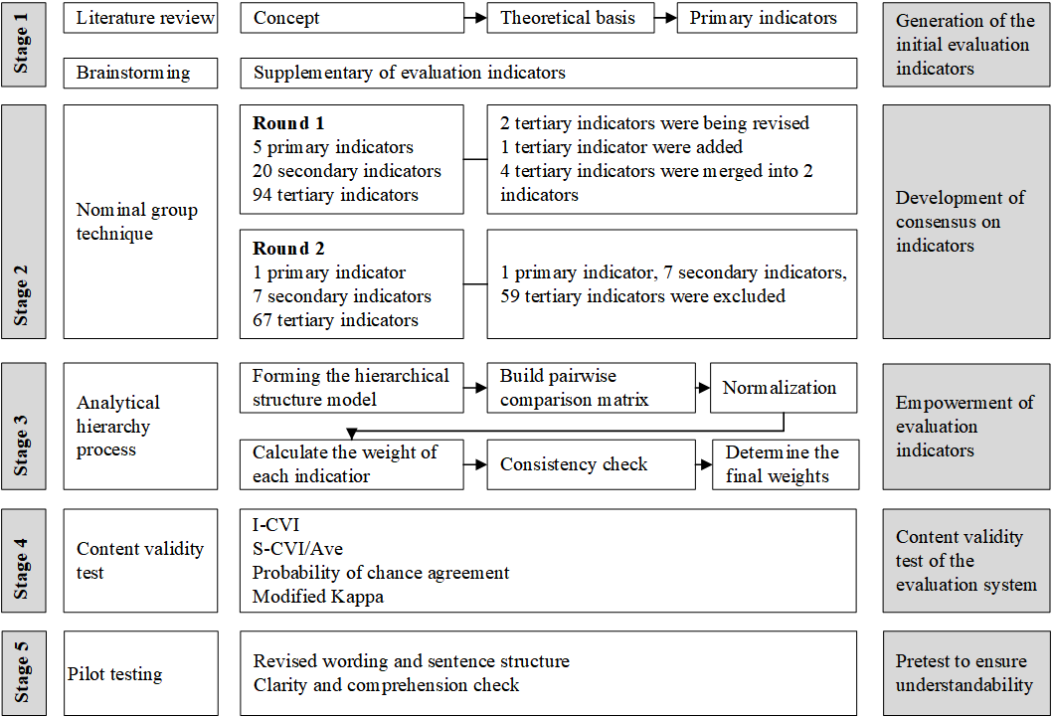
The flowchart for establishing the evaluation system. I-CVI: Item-level Content Validity Index; S-CVI/Ave: Scale-level Content Validity Index, Average.

The overall process of LassVQ development and validation is summarized in Figure 1. This flowchart provides an overview of each step, which is described in detail in the following sections.

### Generation and Categorization of Indicators

Based on the preliminary literature review, a framework was established, including defined dimensions, operational definitions, and an initial item pool. The brainstorming session resulted in the generation of 2 new items and the modification of 3 overlapping items. The final initial version of LassVQ consisted of 5 primary indicators, 20 secondary indicators, and 94 tertiary indicators as illustrated in Appendix S5 in Multimedia Appendix 1.

### Development of Consensus on Indicators

This study adopted 2 rounds of NGT meetings, enrolling a total of 11 experts. Demographic characteristics of the expert panel are outlined in Table 1. Among the selected experts, 2 were male and 9 were female, 45% (5/11) of experts had a doctoral degree. The panel represented a multidisciplinary background, comprising 2 health educational video practitioners, 5 health education researchers, 3 health education practitioners, and 1 library informatics researcher. Furthermore, 80% (9/11) of them ranked associate professor or professor, with 55% (6/11) having more than 20 years of research and work experience. All participating experts were from institutions located in Beijing. The Cs of each key indicator of the NGT was 0.77. The average value of the Ca and Cr was 0.93 and 0.85, respectively. The Kendall W of the revised indicator system is 0.216 (*χ*^2^_49_=105.8, *P*<.001), as presented in Table 2, while our predefined consensus threshold (≥80% experts agreement) was achieved, indicating that consensus could be reached among the experts.

**Table 1.** Demographic characteristics of experts.

Characteristics	Value
Age (y), mean (SD); minimum-maximum	44.82 (7.80); 31‐54
Sex, n (%)	
Male	2 (0.18)
Female	9 (0.82)
Level of education, n (%)	
Bachelor-graduated	3 (0.27)
Postgraduated	3 (0.27)
Doctoral degree	5 (0.45)
Professional background, n (%)	
Health educational videos practitioners	2 (0.18)
Health education researcher	5 (0.45)
Health education practitioner	3 (0.27)
Library informatics researcher	1 (0.09)
Professional title, n (%)	
Senior professor	3 (0.27)
Vice-senior professor	6 (0.55)
Middle title	2 (0.18)
Years of research or work experience, n (%)	
1‐10	2 (0.18)
10‐20	3 (0.27)
>20	6 (0.55)
Degree of authority of experts, mean (SD)	
The degree of familiarity (Cs)	0.77 (0.15)
The coefficient of judgment (Ca)	0.93 (0.08)
The degree of expert opinion authority (Cr)	0.85 (0.10)

**Table 2.** The degree of coordination of experts’ opinions.

Indicator level	W	Chi-square (*df*)	*P* value
Primary indicators	0.23	6.8 (3)	.08
Secondary indicators	0.16	19.5 (12)	.08
Tertiary indicators	0.23	74.3 (32)	<.001
Evaluation system	0.22	105.8 (49)	<.001

In round 1, 11 experts suggested amendments to some indicators. During the discussion, there were 5 primary indicators and 20 secondary indicators, with the number unchanged. The primary indicator, “communication target,” did not reach a consensus. For secondary indicators, 7 of 20 did not reach consensus, which will be addressed in round 2 of NGT. The “reliability” of the primary indicator “communicator” was changed to “credibility.” Tertiary indicators were screened as follows: (1) revised “reasonable and clear navigation structure” to “reasonable navigation structure,” and revised “the comments are positive” to “the positive ratio of comments;” (2) added “views volume;” (3) two indicators “available and easy to use forwarding function” and “available and easy to use like function” were merged into 1 indicator “available and easy to use like and share functions,” and “available and easy to use comments and messages” and “available and easy to use messaging function” were merged into 1 indicator “available and easy to use comments and messages functions.”

In round 2, the expert panel achieved consensus about the final indicator system, which then deleted 1 primary indicator, 7 secondary indicators, and 59 tertiary indicators based on the consensus criteria. The medication and consensus rate of indicators of the 2 rounds of NGT were presented in Appendices S6 and S7 in Multimedia Appendix 1. After the 2 rounds of NGT, 4 primary indicators, 13 secondary indicators, and 34 tertiary indicators finally reached consensus, as presented in Figure 1.

### Empowerment of the Quality Evaluation System

According to the AHP, the quality evaluation system of HESVs on social media was divided into 4 layers. The 9-point intensity of relative weight of importance scale and random index is illustrated in Appendices S2 and S3 in Multimedia Appendix 1. Matrix and weight analysis of the primary indicators (communicator, communication content, communication channel, and communication effect) are presented in Table 3. Among the 4 primary indicators of the evaluation system, “communication content” was perceived as the most influential variable and had a weight of 45.73%, more important than “communicator,” “communication channel,” and “communication effect,” with a relative contribution of 34.58%, 9.93%, and 9.81%, respectively.

**Table 3.** Analysis of the matrix and weight of the primary indicators (consistency index 0.0608).

Primary indicators	(Who) Communicator	(What) Communication content	(In which channel) Communication channel	(With what effect) Communication effect	W	Rank
(Who) Communicator	1	0.21	0.99	0.28	0.10	4
(What) Communication content	4.66	1	4.60	1.32	0.46	1
(In which channel) Communication channel	1.01	0.22	1	0.29	0.10	3
(With what effect) Communication effect	3.52	0.76	3.48	1	0.35	2

The relative weights of secondary indicators are presented in Appendix S8 in Multimedia Appendix 1. The secondary indicators analysis showed that in the “communicator,” “credibility” was perceived as more important than “authoritative” (within-dimensional weight=56.67% and 43.33%, respectively). Among the “communication content,” the relative importance was “scientific” (24.26%), followed by “understandability” (18.59%), “reliability” (18%), “actionability” (14.64%), “watchability” (12.99%), and “attractiveness” (11.53%). In the “communication channel”, “availability” (74.62%) was viewed as more important than “interactivity” (25.38%). Among “communication effect,” the relative importance was “social attention” (39.89%), followed by “perceived usefulness” (36.15%) and “communication engagement” (23.96%).

The relative weights of tertiary indicators are presented in Appendix S8 in Multimedia Appendix 1. The ten tertiary indicators with highest ranks were: (1) recommended as a trending video; (2) positive comments ratio, tied for (3) content’s consistency with scientific common sense, and (4) content complies with ethical standards; (5) content contains necessary relevant information; (6) stable and effective video information source; (7) motivation to share; (8) behavioral guidance or operation techniques are presented with clear steps in logical order; (9) expression is understandable, avoiding medical terms; and (10) theme is clear. The top 1, 2, and 7 tertiary indicators fell into the “communication effect” dimension; numbers 3, 4, 5, 8, 9, and 10 into the “communication content” dimension.

### Content Validity Analysis of the LassVQ Evaluation System

The content validity index for the relevancy of the LassVQ evaluation system was calculated to be 0.87 (Table 4). The kappa designating agreement of relevance of the LassVQ evaluation system was all above 0.74 (κ=0.80‐1.14).

**Table 4.** Content validity index of indicator relevancy and modified kappa of the LassVQ^a^ evaluation system.

Indicator	Experts in agreement	I-CVI^b^	Probability of chance occurrence	Modified kappa
(Who) Communicator	8	0.73	2.90	1.14
(What) Communication content	11	1.00	0.00	1
(In which channel) Communication channel	8	0.73	2.90	1.14
(With what effect) Communication effect	10	0.91	0.01	0.91
Credibility	10	0.91	0.01	0.91
Authoritative	10	0.91	0.01	0.91
Scientific	10	0.91	0.01	0.91
Reliability	11	1.00	0.00	1.00
Attractiveness	11	1.00	0.00	1.00
Watchability	11	1.00	0.00	1.00
Understandability	11	1.00	0.00	1.00
Actionability	11	1.00	0.00	1.00
Availability	11	1.00	0.00	1.00
Interactivity	9	0.82	0.11	0.80
Perceived usefulness	11	1.00	0.00	1.00
Communication engagement	10	0.91	0.01	0.91
Social attention	11	1.00	0.00	1.00
Account’s basic information is disclosed	8	0.73	2.90	1.14
Account’s credibility is high	9	0.82	0.11	0.80
Account’s holder is engaged in medical and health-related domain	10	0.91	0.01	0.91
Account’s platform certification is authoritative	9	0.82	0.11	0.80
Account’s holder has expertise with specialized academic background	9	0.82	0.11	0.80
Content’s consistency with scientific common sense	11	1.00	0.00	1.00
Content complies with ethical standards	10	0.91	0.01	0.91
Content contains visible reference information	7	0.64	92.81	1.00
Content has info that’s kind of needed and related stuff	9	0.82	0.11	0.80
Content aligns with people’s health concerns	11	1.00	0.00	1.00
Content focuses on popular health topics	9	0.82	0.11	0.80
Images, text, and other materials are used properly	11	1.00	0.00	1.00
Image quality is clear	8	0.73	2.90	1.14
Dubbing is clear, louder than background music	7	0.64	92.81	1.00
Dubbing is smooth, without any lag or ambiguity	8	0.73	2.90	1.14
Theme is clear	9	0.82	0.11	0.80
Expression is clear and consistent	10	0.91	0.01	0.91
Expression uses unclear or confusing language	11	1.00	0.00	1.00
Behavioral guidance or operation techniques are presented with clear steps in logical order	9	0.82	0.11	0.80
Photos, graphics, tables, or models are used to explain how to take action clearly	9	0.82	0.11	0.80
Reasonable and clear navigation structure	8	0.73	2.90	1.14
Stable and effective video information source	9	0.82	0.11	0.80
Available and easy to use like and share functions	8	0.73	2.90	1.14
Available and easy to use comments and messages functions	8	0.73	2.90	1.14
Perception of knowledge acquisition	11	1.00	0.00	1.00
Perception of action necessity	10	0.91	0.01	0.91
Intention of behavioral modification	10	0.91	0.01	0.91
Motivation to share	9	0.82	0.11	0.80
Views volume	11	1.00	0.00	1.00
Likes volume	9	0.82	0.11	0.80
Collection volume	8	0.73	2.90	1.14
Repost volume	9	0.82	0.11	0.80
Recommended as a trending video	9	0.82	0.11	0.80
Positive comments ratio	11	1.00	0.00	1.00
Average S-CVI^c^	0.87	—^d^	—	—

a LassVQ: Lasswell’s Video Quality scale

b I-CVI: Item-level Content Validity Index.

c S-CVI: Scale-level Content Validity Index.

d Not applicable.

### Pilot Testing

A total of 15 participants completed the pilot questionnaire, including 9 viewers (aged 19‐46 years) and 6 producers (aged 33‐54 years). Participant characteristics are detailed in Appendix S9 in Multimedia Appendix 1.

The mean readability score across all items was 4.87 (SD 0.26), and the mean comprehensibility score was 4.92 (SD 0.18), indicating that the items were generally clear and understandable. Qualitative feedback highlighted minor issues in wording and phrasing, which were addressed through targeted revisions. For example, vague or informal phrases such as “content has info that’s kind of needed and related stuff” were replaced with clearer wording like “content contains necessary relevant information.” Similarly, ambiguous expressions like “expression uses unclear or confusing language” were revised to “expression is understandable, avoiding medical terms.” These refinements improved the clarity, professionalism, and user-centeredness of the final scale.

## Discussion

### Principal Findings

This study developed LassVQ, an HESVs evaluation system, focusing on 4 key dimensions: communicator, content, channel, and effect, with credibility, scientific rigor, accessibility, and social engagement as core quality indicators. The indicators showed preliminary evidence of acceptable content validity. LassVQ is a preliminary tool that requires further empirical validation. It may, in the future, contribute to supporting researchers in assessing HESVs on social media, aid platforms in video screening, and assist policymakers in developing video production standards.

Valid and reliable measurement tools play an important role in supporting quantitative research and are also frequently used in communication studies [33]. With the emergence and popularity of HESVs on social media and concerns about their quality, it is of both theoretical and practical meaning to explore a quality evaluation system [34]. Based on the 5W model, this research developed the LassVQ evaluation system using the nominal group technique and the analytic hierarchy process. Overall, results of Cs, Ca, and Cr suggested that experts are familiar with the topic, with judgments influenced by practical experience and theoretical analysis and demonstrated a relatively high authority. The result of the modified kappa suggested that the LassVQ evaluation system possesses acceptable content validity [32].

### Comparison With Previous Work

Shawahna [35] and Hong et al [36] used to combine NGT and AHP and facilitated decision-making and quantitatively observe and reviewed in health care dilemmas and streams’ quality, respectively. The NGT calls for methods to include every expert’s opinion. As would be expected, some experts were more vocal than others, and so sessions would include a “round-robin,” which means asking each participant to speak in turn in the order in which they are sitting to ensure each had suggested discussion. The anonymous voting process was intended to encourage participant engagement. The participating experts included both health education researchers and practitioners, along with HESV practitioners and a library informatics researcher. All experts were involved in aspects of HESV production and communication. This combination with AHP allows for analyzing an expert’s subjective judgment with mathematical form and conducting multiobjective decision-making analysis of the scientific treatment to ensure the scientific result [37]. Experts adopt judgment matrices to determine the importance value of each indicator, and the result shows that the consistency of the pairwise comparison matrices at all levels met the consistency test criterion, with CR<0.10 [37], suggesting acceptable logical consistency and supporting scientific rigor and reliability. The weighted value of the primary indicator was in the following order: communication content (0.46), communication effect (0.34), communication channel (0.10), and communicator (0.10). Existing tools were designed for printed materials, developed many years ago, and may not be fully applicable to the social media context. Furthermore, DISCERN consists of 6 questions focusing on diseases and treatment, while the JAMA benchmarks take advertisement into consideration.

The Lasswell’s 5W communication model, a classic theory in information communication, offers a theoretical framework for understanding the dissemination process of HESVs on social media [25]. It was put forward in 1948 and still has its applicability to this day, which divides the communication process into 5 parts, including the communicator, information, channel, audience, and effect. Critics argue that the 5W model’s linear approach is outdated and insufficient for adapting to the dynamic environment of the internet [38]. However, consistent with the point by Sapienza et al [39], this study holds that the 5W model is flexible and we should dialectically explore new applications of it. For instance, Wei et al [24] expanded the 5W model into an 8-element communication framework for internet health rumors, while Xu et al [40] designed a questionnaire based on the 5W model to investigate caregivers’ needs regarding medication content and dissemination channels. In the 2 rounds of NGT discussions and indicator evaluations, this study removed the audience dimension from the original 5W model and restructured the dimensions into 4 categories: communicator, communication content, communication channel, and communication effect. Experts concurred that within the social media context, the roles of communicator and audience often overlap, with individuals potentially fulfilling both roles simultaneously [25]. Since audience research typically examines viewer characteristics and responses, while our study focuses on the quality of HESVs, including this dimension was considered outside our methodological scope.

Following the NGT discussions, the study excluded the communicator’s influence, originally measured by tertiary indicators such as the communicator’s total number of followers, total number of received likes, and total number of posted videos. Bengi et al [41] advised focusing on the communicator’s credibility and professional background rather than metrics like follower count or video quantity. The elaboration likelihood model indicated that professional titles may serve as signals of expertise, potentially enhancing viewer engagement [42,43]. The influence of the communication channel was also removed, including metrics such as “number of platform users,” “frequency of platform being forwarded by mainstream media,” and “the proportion of health popularization videos on the platform.” While platforms like TikTok (ByteDance) garner significantly more views and likes than YouTube, they have comparatively fewer high-quality videos [41]. The model also eliminated breadth-of-spread metrics, such as “balanced gender of users,” “balanced age distribution of users,” “wide distribution of users,” and “balanced classification of the user’s city.” The NGT experts argued that these indicators were considered of limited significance and that data availability was relatively low. When producing or evaluating HESVs, priority should be given to the content’s theme and target audience [15,44]. For example, videos about menstruation inherently tend to attract an audience with an imbalanced gender and age distribution. For “communication recognition” and “communication participation,” NGT experts emphasized the significance of views as a direct communication metric. They also suggested combining views, likes, collections, and reposts as a single metric, which aligns with the research by Erdem and Karaca [45] on the video power index used for evaluating YouTube content.

For the tertiary indicators of the understandability of communication content, the NGT removed some indicators deemed less relevant. Among those addressing the consistency between theme and title, only “theme is clear” was retained as the most representative indicator [46]. Indicators like informative subtitles, clear summaries, 3 to 5 core points, and highlighting of core information were also removed, as they were deemed unsuitable in the context of short social media videos [18]. This study refined the tertiary indicators for content attractiveness by focusing on the “three Vs” of communication—verbal, vocal, and visual [16], a model recommended for enhancing educational outcomes in HESVs [18]. Experts noted that the indicator “subtitles are consistent with dubs” was inappropriate since subtitles might be autogenerated text. Indicators of actionability were narrowed down to emphasize logical presentation and clear guidance on how to take action, consistent with the study by Sui and Zhang [47] on the role of logical coherence in changing attitudes. All indicators related to perceived cultural appropriateness were excluded, as experts argued that evaluating HESVs based on target audience and linguistic preferences might lead to conflicting conclusions.

### Strengths and Limitations

This study is the first to develop a comprehensive evaluation system for HESVs on social media (LassVQ). Some limitations of this study should be noted. The NGT itself is less commonly used than the Delphi process in health communication, with disadvantages including limited rounds of voting [48]. However, this is outweighed by allowing face-to-face discussions and anonymous voting, which yield higher response rates than would have been achieved with mailed questionnaires [49]. Further research is required to confirm the validity of the findings obtained here on this evaluation system by applying it to HESVs on social media platforms. For the pilot study, the small sample size limits the generalizability of the findings related to readability and comprehensibility. As an exploratory step, the pilot was primarily intended to guide item refinement, and its results should be interpreted with caution. The expert selection process, constrained by the authors’ available resources, may introduce certain limitations and potential biases. The other limitation of the study lies in its focus on short-form video content, which may limit the applicability of the findings to other types of media, such as long-form videos or text-based health information. Although short videos are highly popular on social media, the evaluation criteria developed may not fully capture the nuances of different content formats. Future research could explore how the evaluation system might need to be adapted or expanded for other forms of health communication content across various platforms. The LassVQ evaluation system may offer a useful framework for analyzing the current circumstance of HESVs on social media, although further application and validation are needed to confirm its broader utility.

### Further Directions

A more detailed investigation into the key factors for the quality improvement of HESVs on social media is needed to clarify. To support external validation, several health video producers and viewers will apply the LassVQ to a representative sample of high-view HESVs from major social media platforms. Intraclass correlation coefficients will be used to evaluate interrater reliability. Videos will also be rated using DISCERN and Global Quality Score, enabling correlation analyses to examine LassVQ’s predictive accuracy.

### Conclusions

In summary, the quality evaluation system for HESVs on social media (LassVQ) developed in this study offers a structured approach to enhancing the quality of health communication throughout the entire video production and dissemination process. The system equips health care professionals with a research-based tool for the design, production, and evaluation of HESVs, with an emphasis on critical quality indicators. Future research could expand on these findings by refining the evaluation criteria, integrating additional perspectives from health care professionals, or tailoring the system to address specific health topics. The application of the LassVQ enables researchers and practitioners to continually enhance the effectiveness and impact of health education content disseminated on social media platforms.

## Supplementary material

10.2196/72661 Multimedia Appendix 1 Materials related to the development and validation of the Lasswell’s Video Quality scale (LassVQ) evaluation system.

10.2196/72661 Checklist 1 COSMIN checklist.

## Abbreviations

5W: Lasswell’s 5W communication model
AHP: analytic hierarchy process
COSMIN: Consensus-Based Standards for the Selection of Health Status Measurement Instruments
HESV: health educational short video
I-CVI: Item-level Content Validity Index
*JAMA*: *Journal of the American Medical Association*
LassVQ: Lasswell’s Video Quality scale
NGT: nominal group technique
S-CVI/Ave: Scale-level Content Validity Index, Average

## References

[1] Xiao L, Min H, Wu Y (2023). Public’s preferences for health science popularization short videos in China: a discrete choice experiment. Front Public Health.

[2] Moorhead SA, Hazlett DE, Harrison L, Carroll JK, Irwin A, Hoving C (2013). A new dimension of health care: systematic review of the uses, benefits, and limitations of social media for health communication. J Med Internet Res.

[3] Gottfried J (2024). Americans’ social media use. Pew Research Center.

[4] Kemp S Digital 2024: China. DataReportal.

[5] Mohamed F, Shoufan A (2024). Users’ experience with health-related content on YouTube: an exploratory study. BMC Public Health.

[6] Gabarron E, Larbi D, Dorronzoro E, Hasvold PE, Wynn R, Årsand E (2020). Factors engaging users of diabetes social media channels on Facebook, Twitter, and Instagram: observational study. J Med Internet Res.

[7] Loh YL, Yaw QP, Lau Y (2023). Social media-based interventions for adults with obesity and overweight: a meta-analysis and meta-regression. Int J Obes.

[8] Goodyear VA, Boardley I, Chiou SY (2021). Social media use informing behaviours related to physical activity, diet and quality of life during COVID-19: a mixed methods study. BMC Public Health.

[9] Noetel M, Griffith S, Delaney O (2021). Video improves learning in higher education: a systematic review. Rev Educ Res.

[10] Suarez-Lledo V, Alvarez-Galvez J (2021). Prevalence of health misinformation on social media: systematic review. J Med Internet Res.

[11] Vosoughi S, Roy D, Aral S (2018). The spread of true and false news online. Science.

[12] Bizzotto N, de Bruijn GJ, Schulz PJ (2023). Buffering against exposure to mental health misinformation in online communities on Facebook: the interplay of depression literacy and expert moderation. BMC Public Health.

[13] Thapa DK, Visentin DC, Kornhaber R, West S, Cleary M (2021). The influence of online health information on health decisions: a systematic review. Patient Educ Couns.

[14] Helming AG, Adler DS, Keltner C, Igelman AD, Woodworth GE (2021). The content quality of YouTube videos for professional medical education: a systematic review. Acad Med.

[15] Charnock D (1998). The DISCERN Handbook: Quality Criteria for Consumer Health Information on Treatment Choices.

[16] Silberg WM, Lundberg GD, Musacchio RA (1997). Assessing, controlling, and assuring the quality of medical information on the internet: Caveant lector et viewor--let the reader and viewer beware. JAMA.

[17] Boyer C, Selby M, Appel RD (1998). The Health On the Net Code of Conduct for medical and health web sites. Stud Health Technol Inform.

[18] Azer SA (2020). Are DISCERN and JAMA suitable instruments for assessing YouTube videos on thyroid cancer? Methodological concerns. J Cancer Educ.

[19] Shoemaker SJ, Wolf MS, Brach C (2014). Development of the Patient Education Materials Assessment Tool (PEMAT): a new measure of understandability and actionability for print and audiovisual patient information. Patient Educ Couns.

[20] Huang MM, Winoker JS, Allaf ME, Matlaga BR, Koo K (2021). Evidence-based quality and accuracy of YouTube videos about nephrolithiasis. BJU Int.

[21] Charnock D, Shepperd S, Needham G, Gann R (1999). DISCERN: an instrument for judging the quality of written consumer health information on treatment choices. J Epidemiol Community Health.

[22] Cortes Cavalcante J, Faria Sales M, Sousa Junior R de (2024). Analysis of the Brazilian-Portuguese content on autism spectrum disorder available on YouTube videos. Phys Occup Ther Pediatr.

[23] Fat MJL, Doja A, Barrowman N, Sell E (2011). YouTube videos as a teaching tool and patient resource for infantile spasms. J Child Neurol.

[24] Wei H, Chen J, Gan X, Liang Z (2022). Eight-element communication model for internet health rumors: a new exploration of Lasswell’s “5W communication model”. Healthcare (Basel).

[25] Peng W (2015). Analysis of new media communication based on Lasswell’s “5W” model. Model Journal of Educational and Social Research.

[26] Liberatore MJ, Nydick RL (2008). The analytic hierarchy process in medical and health care decision making: a literature review. Eur J Oper Res.

[27] Gagnier JJ, de Arruda GT, Terwee CB, Mokkink LB, Consensus group (2025). COSMIN reporting guideline for studies on measurement properties of patient‑reported outcome measures: version 2.0. Qual Life Res.

[28] (1998). Consensus development methods, and their use in clinical guideline development. Health Technol Assess.

[29] Nair R, Aggarwal R, Khanna D (2011). Methods of formal consensus in classification/diagnostic criteria and guideline development. Semin Arthritis Rheum.

[30] Saaty TL (2005). Theory and Applications of the Analytic Network Process: Decision Making With Benefits, Opportunities, Costs, and Risks.

[31] Forman EH, Gass SI (2001). The analytic hierarchy process—an exposition. Oper Res.

[32] Polit DF, Beck CT, Owen SV (2007). Is the CVI an acceptable indicator of content validity? Appraisal and recommendations. Res Nurs Health.

[33] Rubin RB, Palmgreen P, Sypher HE (2020). Communication Research Measures.

[34] Zhao SS, Zhang BQ, Chang XH (2023). iQiYi video as a source of information on COVID-19 vaccine: content analysis. Disaster Med Public Health Prep.

[35] Shawahna R (2020). Facilitating ethical, legal, and professional deliberations to resolve dilemmas in daily healthcare practice: a case of driver with breakthrough seizures. Epilepsy Behav.

[36] Hong CY, Chung ES, Chang H (2020). The right to urban streams: quantitative comparisons of stakeholder perceptions in defining adaptive stream restoration. Sustainability.

[37] Saaty RW (1987). The analytic hierarchy process—what it is and how it is used. Mathematical Modelling.

[38] McQuail D (1985). Sociology of Mass Communication. Annu Rev Sociol.

[39] Sapienza ZS, Iyer N, Veenstra AS (2015). Reading Lasswell’s model of communication backward: three scholarly misconceptions. Mass Communication and Society.

[40] Xu X, Wang Z, Li X (2022). Acceptance and needs of medication literacy education among children by their caregivers: a multicenter study in mainland China. Front Pharmacol.

[41] Bengi VU, Saraç Atagün Ö, Ceylan Şen S, Özcan E, Paksoy T, Güler ÖŞ (2024). How much information regarding gingival enlargement can we get from TikTok and YouTube?. Spec Care Dentist.

[42] Tan Y, Geng S, Chen L, Wu L (2023). How doctor image features engage health science short video viewers? Investigating the age and gender bias. IMDS.

[43] Zha X, Yang H, Yan Y, Liu K, Huang C (2018). Exploring the effect of social media information quality, source credibility and reputation on informational fit-to-task: Moderating role of focused immersion. Comput Human Behav.

[44] (2019). CDC Clear Communication Index: a tool for developing and assessing CDC public communication products. Centers for Disease Control and Prevention.

[45] Erdem MN, Karaca S (2018). Evaluating the accuracy and quality of the information in kyphosis videos shared on YouTube. Spine (Phila Pa 1976).

[46] (2023). How to develop products for adults with intellectual developmental disabilities and extreme low literacy: a product development tool. https://www.cdc.gov/ccindex/pdf/idd-ell-product-development-tool-508.pdf.

[47] Sui Y, Zhang B (2021). Determinants of the perceived credibility of rebuttals concerning health misinformation. Int J Environ Res Public Health.

[48] Humphrey-Murto S, Varpio L, Wood TJ (2017). The use of the Delphi and other consensus group methods in medical education research: a review. Acad Med.

[49] Black N, Murphy M, Lamping D (1999). Consensus development methods: a review of best practice in creating clinical guidelines. J Health Serv Res Policy.

