# Supplementary material for: Developing an Evaluation System for Quality of Health Educational Short Videos on Social Media (LassVQ) Using Nominal Group Technique and Analytic Hierarchy Process: Qualitative Study
Source: J Med Internet Res. 2025 Sep 18;27:e72661. doi: 10.2196/72661 (PMC12445618; doi:10.2196/72661)
Supplement: Multimedia Appendix 1 [file jmir-v27-e72661-s001.docx]

# Appendix S1. Literature review protocol

**Research question:**

What are the quality evaluation indicators for health educational short videos on social media?

**Approach:**

A structured literature review of indicators or factors identified in the scientific literature for evaluating the quality of health educational short videos on social media. Both structured reviews and original research—published as peer-reviewed journal articles, academic reports, or conference proceedings—were included if they were relevant to the development or application of evaluation indicators.

**Databases:**

Pubmed, Web of Science (Web of Science Core Collection), Embase (Embase, MEDLINE, Preprints, Clinical Trials), CNKI （中国知网——学术期刊、学位论文）, WANFANG （万方数据知识服务平台）

**Concepts:**

**- Social media**: social media, social networking, YouTube, TikTok, Douyin, Facebook, Twitter, Kuai, Bilibili, Weibo, WeChat

**- Video:** video

**- Quality:** quality, reliab*, understandability, actionab*, credib*, interactiv*, comprehensive*, accuracy, completeness, clarity

**Inclusion criteria:**

Full text available

Publications dated from database inception to May 2024

Only full text articles (no conference abstracts)

Both structured reviews (e.g., systematic reviews, scoping reviews) and original research articles

Scientific articles that discuss determinants of health educational short videos on social media

Written in English or Chinese

**Exclusion criteria:**

Duplicate publications

Studies that assess only content accuracy without mentioning quality evaluation indicators or frameworks

Editorials, commentaries, letters to the editor, conference abstracts, or protocols without empirical information

**Search strategy**

**PubMed**

| **A: Social Media** | | |
| --- | --- | --- |
| Concept | MeSH term | Free text (searched in title and abstract) |
| Social Media | “Social Media” | social media, social networking, YouTube, TikTok, Douyin, Kuai, Bilibili |
| Search query | (social media[MeSH Terms]) OR ("social media"[Title/Abstract] OR "social networking"[Title/Abstract] OR "YouTube"[Title/Abstract] OR "TikTok"[Title/Abstract] OR "Douyin"[Title/Abstract] OR "Facebook"[Title/Abstract] OR "Twitter"[Title/Abstract] OR "Kuai"[Title/Abstract] OR "Bilibili"[Title/Abstract] OR "Weibo"[Title/Abstract] OR "WeChat"[Title/Abstract]) | |
| **B: Video** | | |
| Concept | MeSH term | Free text (searched in title and abstract) |
| Video | / | video |
| Search query | video[Title/Abstract] | |
| **C: Quality** | | |
| Concept | MeSH term | Free text (searched in title and abstract) |
| Quality | / | quality, reliab*, understandability, actionab*, credib*, interactiv*, comprehensive*, accuracy, completeness, clarity |
| Search query | ("quality"[Title/Abstract] OR "reliab*"[Title/Abstract] OR "understandability"[Title/Abstract] OR "actionab*"[Title/Abstract] OR "credib*"[Title/Abstract] OR "interactiv*"[Title/Abstract] OR "comprehensive*"[Title/Abstract] OR "accuracy"[Title/Abstract] OR "completeness"[Title/Abstract] OR "clarity"[Title/Abstract]) | |

**Complete query:** (((social media[MeSH Terms]) OR ("social media"[Title/Abstract] OR "social networking"[Title/Abstract] OR "YouTube"[Title/Abstract] OR "TikTok"[Title/Abstract] OR "Douyin"[Title/Abstract] OR "Facebook"[Title/Abstract] OR "Twitter"[Title/Abstract] OR "Kuai"[Title/Abstract] OR "Bilibili"[Title/Abstract] OR "Weibo"[Title/Abstract] OR "WeChat"[Title/Abstract])) AND ("quality"[Title/Abstract] OR "reliab*"[Title/Abstract] OR "understandability"[Title/Abstract] OR "actionab*"[Title/Abstract] OR "credib*"[Title/Abstract] OR "interactiv*"[Title/Abstract] OR "comprehensive*"[Title/Abstract] OR "accuracy"[Title/Abstract] OR "completeness"[Title/Abstract] OR "clarity"[Title/Abstract])) AND (video[Title/Abstract])

**Results:** 1864 (10/5/2024)

**Web of Science (Web of Science Core Collection)**

#1: TS=("social media" OR "social networking" OR "YouTube" OR "TikTok" OR "Douyin" OR "Facebook" OR "Twitter" OR "Kuai" OR "Bilibili" OR "Weibo" OR "WeChat")

#2: TS=(video)

#3: TS=("quality" OR "reliab*" OR "understandability" OR "actionab*" OR "credib*" OR "interactiv*" OR "comprehensive*" OR "accuracy" OR "completeness" OR "clarity")

#4: #1 AND #2 AND #3

**Complete query:** ((TS=("social media" OR "social networking" OR "YouTube" OR "TikTok" OR "Douyin" OR "Facebook" OR "Twitter" OR "Kuai" OR "Bilibili" OR "Weibo" OR "WeChat")) AND TS=(video)) AND TS=("quality" OR "reliab*" OR "understandability" OR "actionab*" OR "credib*" OR "interactiv*" OR "comprehensive*" OR "accuracy" OR "completeness" OR "clarity")

**Results:** 6191 (10/5/2024)

**Embase** **(Embase, MEDLINE, Preprints, Clinical Trials)**

| **A: Social Media** | | |
| --- | --- | --- |
| Concept | Emtree term | Free text (searched in title, abstract, and author keywords) |
| Social Media | “Social Media” | social media, social networking, YouTube, TikTok, Douyin, Kuai, Bilibili |
| Search query | 'social media':ti,ab,kw OR 'social networking':ti,ab,kw OR 'youtube':ti,ab,kw OR 'tiktok':ti,ab,kw OR 'douyin':ti,ab,kw OR 'facebook':ti,ab,kw OR 'twitter':ti,ab,kw OR 'kuai':ti,ab,kw OR 'bilibili':ti,ab,kw OR 'weibo':ti,ab,kw OR 'wechat':ti,ab,kw | |
| **B: Video** | | |
| Concept | Emtree term | Free text (searched in title, abstract, and author keywords) |
| Video | / | video |
| Search query | video:ti,ab,kw | |
| **C: Quality** | | |
| Concept | Emtree term | Free text (searched in title, abstract, and author keywords) |
| Quality | / | quality, reliab*, understandability, actionab*, credib*, interactiv*, comprehensive*, accuracy, completeness, clarity |
| Search query | 'quality':ti,ab,kw OR 'reliab*':ti,ab,kw OR 'understandability':ti,ab,kw OR 'actionab*':ti,ab,kw OR 'credib*':ti,ab,kw OR 'interactiv*':ti,ab,kw OR 'comprehensive*':ti,ab,kw OR 'accuracy':ti,ab,kw OR 'completeness':ti,ab,kw OR 'clarity':ti,ab,kw | |

**Complete query:** video:ti,ab,kw AND ('quality'/exp OR 'quality' OR 'quality':ti,ab,kw OR 'reliab*':ti,ab,kw OR 'understandability':ti,ab,kw OR 'actionab*':ti,ab,kw OR 'credib*':ti,ab,kw OR 'interactiv*':ti,ab,kw OR 'comprehensive*':ti,ab,kw OR 'accuracy':ti,ab,kw OR 'completeness':ti,ab,kw OR 'clarity':ti,ab,kw) AND ('social media'/exp OR 'social media' OR 'social media':ti,ab,kw OR 'social networking':ti,ab,kw OR 'youtube':ti,ab,kw OR 'tiktok':ti,ab,kw OR 'douyin':ti,ab,kw OR 'facebook':ti,ab,kw OR 'twitter':ti,ab,kw OR 'kuai':ti,ab,kw OR 'bilibili':ti,ab,kw OR 'weibo':ti,ab,kw OR 'wechat':ti,ab,kw)

**Results:** 2817 (10/5/2024)

**CNKI （中国知网——学术期刊、学位论文）**

**Complete query:**（篇关摘：社交媒体 + 社交网络 + YouTube + 抖音 + 脸书 + 推特 + 快手 + 哔哩哔哩 + 微博 + 微信(精确)）AND（篇关摘：视频(精确)）AND（篇关摘：质量 + 可靠性 + 可理解性 + 可操作性 + 可信度 + 交互性 + 全面性 + 准确性 + 完整性 + 清晰度(精确)）

**Results:** 3543 (10/5/2024)

**WANFANG （万方数据知识服务平台）**

**Complete query:** 题名或关键词:(社交媒体 OR 社交网络 OR YouTube OR 抖音 OR 脸书 OR 推特 OR 快手 OR 哔哩哔哩 OR 微博 OR 微信 ) and 题名或关键词:(视频) and 题名或关键词:(质量 OR 可靠性 OR 可理解性 OR 可操作性 OR 可信度 OR 交互性 OR 全面性 OR 准确性 OR 完整性 OR 清晰度)

**Results:** 411 (10/5/2024)

**Literature review PRISMA flow diagram**

## Identification

## Eligibility

## Included

## Screening

Records identified through database searching (n = 14826)

- PubMed (n = 1864)
- Web of Science (n = 6191)
- Embase (n = 2817)
- CNKI (n = 3543)
- WANFANG (n = 411)

Duplicates removed
(n = 5401)

## Records excluded (n = 9119)

## Not relevant (n = 8539)

## Wrong language (n = 158)

## No full text available (n = 137)

## Conference abstract (n = 285)

Records screened on title and abstract
(n = 9425)

Full-text articles excluded
(n = 242)

Full-text articles assessed for eligibility
(n = 306)

33)

Additional records included through previous related review (n = 6) and additional search update (n = 2)

Studies included in qualitative synthesis
(n = 72)

**The initial indicator system and main references**

| Primary Indicator | Secondary Indicator | Tertiary Indicator | Main References |
| --- | --- | --- | --- |
| Communicator | Reliability | Disclosure of basic account information | [1, 2] |
|  |  | High account credibility | [3] |
|  |  | Health science popularization as the primary motivation for publishing | [4-7] |
|  |  | Account holder is engaged in the healthcare or medical field | [2] |
|  |  | Content primarily aims to improve public health literacy | [6] |
|  | Influence | Total number of followers | [8-10] |
|  |  | Total number of likes | [11] |
|  |  | Total number of videos published | [12] |
|  | Authority | Authoritative account type | [1, 4, 5, 9, 13-17] |
|  |  | Official platform verification | [2, 18] |
|  |  | Possesses adequate professional knowledge related to the topic | [1, 2, 8] |
| Content | Scientific Accuracy | Content aligns with modern medical consensus and scientific facts | [19-24] |
|  |  | Content reflects proper political and ethical orientation | [24] |
|  |  | Privacy protection is ensured | [24, 25] |
|  |  | Absence of biased information | [2, 26, 27] |
|  |  | Content is based on objective facts, not personal opinion | [28] |
|  |  | Content is original to some extent | [29] |
|  | Trustworthiness | Creator's identity is fully disclosed | [2, 13, 30-32] |
|  |  | Sources of information are cited | [2, 8, 15, 26, 33] |
|  |  | Free from advertisements or potential conflicts of interest | [34] |
|  |  | Includes necessary supporting information | [2, 35] |
|  |  | Evidence is provided to support the content | [35, 36] |
|  | Attractiveness | Video descriptions are appealing | [8, 22] |
|  |  | Video thumbnails are engaging | [8, 37] |
|  |  | Content addresses public health concerns | [27] |
|  |  | Content relates to trending topics at the time of release | [1, 2, 38, 39] |
|  |  | Descriptions clearly highlight the value or relevance of the video content | [8] |
|  |  | Entertaining and educational design | [33, 39] |
|  | Watchability | Appropriate use of visuals and text | [33] |
|  |  | Suitable duration | [40, 41] |
|  |  | Clear image quality | [19] |
|  |  | Aesthetic color scheme | [41] |
|  |  | Voice-over is clear and louder than background music | [19] |
|  |  | Smooth and unambiguous narration | [19, 33] |
|  |  | Appropriate use of music and sound effects | [33, 42] |
|  |  | Suitable filming and editing techniques | [33, 42] |
|  |  | Rich variety in production format | [43] |
|  |  | Clear subtitles | [8, 22, 44] |
|  | Comprehensibility | Clear main theme | [8, 22, 45, 46] |
|  |  | Title aligns with the theme | [46, 47] |
|  |  | Title length is appropriate | [7, 21, 41, 48] |
|  |  | Thumbnail matches the video content | [8, 47] |
|  |  | Clearly defined target audience | [46] |
|  |  | Concise and non-redundant information | [25] |
|  |  | Informative subtitles | [49] |
|  |  | Conclusion presented for videos over 3 minutes | [45] |
|  |  | 3–5 core messages included | [22, 25] |
|  |  | Core messages are highlighted | [22] |
|  |  | Visual cues used for key information | [45] |
|  |  | Clear and consistent language references | [50] |
|  |  | Characters appear consistently throughout the video | [2, 19, 50] |
|  |  | Narration aligns with visuals | [22] |
|  |  | Narration matches subtitles | [51] |
|  |  | Clear explanation of technical terms | [7, 22, 52] |
|  |  | Language is simple and easy to understand | [7, 33] |
|  |  | Clear explanation of numerical values, units, and calculations | [7, 46] |
|  |  | Use of photos, images, tables, or models to support content | [19, 49] |
|  | Actionability | Provides practical medical advice, behavior guidance, or operational skills | [45, 53, 54] |
|  |  | Advice or guidance is directed at the target audience | [46] |
|  |  | Guidance is presented in a logical order with clear steps | [22] |
|  |  | Clear explanation on how to use photos, images, tables, or models to take action | [46] |
| Dissemination Channel | Accessibility | Clear and logical navigation structure | [2, 29, 55] |
|  |  | Comprehensive search results | [56] |
|  |  | Accurate search results | [56, 57] |
|  |  | Stable and valid video source | [2, 56] |
|  | Interactivity | Easy-to-use sharing function | [2, 13] |
|  |  | Easy-to-use like function | [2] |
|  |  | Easy-to-use comment function | [2] |
|  |  | Easy-to-use private messaging function | [2] |
|  | Influence | Number of platform users | [12] |
|  |  | Frequency of reposts by mainstream media | [12, 58] |
|  |  | Proportion of health science content on the platform | [12] |
|  | Authority | Clear and strict video review mechanism | [42] |
|  |  | Number of health-certified accounts on the platform | [12] |
| Audience | Perceived Cultural Appropriateness | Video respects your customs | [59] |
|  |  | Video respects your cultural traditions | [59] |
|  |  | Video respects your language habits | [5] |
|  | Perceived Usefulness | Video provides health science information | [13, 60-62] |
|  |  | Video meets your health information needs | [63] |
|  |  | Video motivates you to take action | [61] |
|  |  | You would change your behavior based on the video | [62] |
|  |  | You would recommend the video to others | [64, 65] |
|  | Perceived Ease of Use | You can access the health information in the video | [39, 62] |
|  |  | You can evaluate the reliability of the health information in the video | [61] |
|  |  | You can understand the health information in the video | [2, 60, 61] |
|  |  | You can apply the health information in the video | [61] |
| Communication Effects | Breadth of Reach | Balanced gender distribution among users | [66-68] |
|  |  | Wide geographical distribution of users | [25] |
|  | Recognition | Number of likes | [58, 67, 69] |
|  |  | Number of saves/favorites | [8, 70] |
|  | Engagement | Number of comments | [70, 71] |
|  |  | Number of shares | [58, 67] |
|  | Public Attention | Featured on platform’s trending list | [70] |
|  |  | Reposted by official media or professional health science platforms | [8] |
|  |  | Positive and active comments or bullet chats | [70, 72] |

# Appendix S2. Impact degree scores of each judgment basis.

| Judgment bases | Impact degree scores of expert judgments | | |
| --- | --- | --- | --- |
|  | large | middle | little |
| Practical experience | 0.5 | 0.4 | 0.3 |
| Theoretical analysis | 0.3 | 0.2 | 0.1 |
| Reference to domestic and foreign counterparts | 0.1 | 0.1 | 0.1 |
| Intuition | 0.1 | 0.1 | 0.1 |

# Appendix S3. Saaty’s 9-point intensity of relative importance weight scale.

| Intensity of importance/well-being | Definition | Significance |
| --- | --- | --- |
| 1 | Equal importance/equally good | Two activities contribute equally to objective |
| 3 | Moderate importance of one factor over another/weakly | Experience and judgment slightly favor one activity over another |
| 5 | Strong or essential importance/strongly | Experience and judgment strongly favor one activity over another |
| 7 | Very strong importance/very strongly | An activity is strongly favored, and its dominance is demonstrated in practice |
| 9 | Extreme importance/absolutely better | The evidence favoring one activity over another is of the highest possible order of affirmation |
| 2,4, 6,8 | Intermediate value between the two adjacent judgments | When a compromise is needed |

# Appendix S4. Random index (RI).

| n | 1 | 2 | 3 | 4 | 5 | 6 | 7 | 8 | 9 | 10 | 11 | 12 | 13 | 14 | 15 |
| --- | --- | --- | --- | --- | --- | --- | --- | --- | --- | --- | --- | --- | --- | --- | --- |
| RI | 0 | 0 | 0.58 | 0.9 | 1.12 | 1.24 | 1.32 | 1.41 | 1.45 | 1.49 | 1.52 | 1.54 | 1.56 | 1.58 | 1.59 |

# Appendix S5. Initial evaluation indicators of LassVQ.

| Primary indicator | Secondary indicator | Tertiary indicator |
| --- | --- | --- |
| Communicator | Reliability | Account basic information disclosure |
|  |  | Account credibility |
|  |  | Whether the account's motivation for posting is health knowledge |
|  |  | Whether the account holder is engaged in medical and health-related industries |
|  | Influence | Account's total number of followers |
|  |  | Account's total number of received likes |
|  |  | Account's total number of posted videos |
|  | Authoritative | Account type's authority |
|  |  | Account's authentication credentials |
|  |  | Account's subject-related expertise |
| Communication content | Scientific | Content's consistency with modern medical consensus and scientific common sense |
|  |  | Content's political orientation, value orientation and complies with ethical standards |
|  |  | Attention to privacy |
|  |  | Unbiased information |
|  |  | The content is objective facts, not personal opinions |
|  |  | The content has a certain degree of originality |
|  | Reliability | The content's creator's information is fully public |
|  |  | Reference information source is open |
|  |  | No ads or potentially harmful information |
|  |  | Content has info that’s kind of needed and related stuff |
|  |  | Content contains supported evidence |
|  | Attractiveness | Attractive video description |
|  |  | Attractive video cover design |
|  |  | Content is in line with public health concerns |
|  |  | Content focuses on hot topics that were popular when the video was released |
|  |  | Content design is both entertaining and educational, and interesting |
|  | Watchability | Proper use of images, text and other materials |
|  |  | Suitable duration |
|  |  | Clear image quality |
|  |  | Vivid and beautiful colors |
|  |  | Clear dubbing, with the louder volume than the background music |
|  |  | Smooth dubbing, without any lag or ambiguity |
|  |  | Appropriate use of music and sound effects |
|  |  | Suitable shooting and post-production techniques |
|  |  | Rich creative forms |
|  |  | Clear subtitles |
|  | Understandability | Clear theme |
|  |  | Have a title that matches the subject |
|  |  | The title should have an appropriate number of words |
|  |  | The cover is consistent with the subject content |
|  |  | Have clear target users |
|  |  | Concise and not redundant information |
|  |  | Have informative subtitles |
|  |  | Videos longer than 3 minutes have a clear summary at the end |
|  |  | The number of core information is 3 to 5 |
|  |  | Core information highlights |
|  |  | Use visual cues for key information |
|  |  | The language is clear and consistent |
|  |  | The characters in the video are consistent |
|  |  | Consistent dubbing and the content of the picture |
|  |  | Consistent dubbing and subtitles |
|  |  | Expression uses unclear or confusing language |
|  |  | Understandable language |
|  |  | Clear explanation for the meaning of numbers, units and mathematical operations |
|  |  | Photos, images, tables or models to support popular science content |
|  | Actionability | Provide practical medical advice, behavioural guidance or operation technology |
|  |  | Direct medical advice, behavioural guidance, or operational techniques to the target audience |
|  |  | Present the popular science content of behavioural guidance or operation techniques in a logical order with clear steps |
|  |  | Clearly explain how to take action using photos, graphics, tables or models |
| Communication channel | Availability | Reasonable and clear navigation structure |
|  |  | Search results comprehensiveness |
|  |  | Search results accuracy |
|  |  | Stable and effective video information source |
|  | Interactivity | Available and easy to use forwarding function |
|  |  | Available and easy to use like function |
|  |  | Available and easy to use comments and messages |
|  |  | Available and easy to use messaging function |
|  | Influence | Number of platform users |
|  |  | Frequency of platform being forwarded by mainstream media |
|  |  | The proportion of health popularization videos on the platform |
|  | Authoritative | The platform has a clear and strict video review mechanism |
|  |  | Number of health certification accounts on the platform |
| Communication target | Perceived cultural appropriateness | Video respects your custom background |
|  |  | Video respects your cultural traditions |
|  |  | Video respects your language habits |
|  | Perceived usefulness | Video provides you with health science knowledge |
|  |  | Video meets your health information needs |
|  |  | Video makes you feel the need to take action |
|  |  | You will change your behaviour based on this video |
|  |  | You'd like to recommend this video to others |
|  | Perceived ease of use | You can get health information in the video |
|  |  | You can understand the health information in the video |
|  |  | You can apply the health information in the video |
| Communication effect | Spread | Balanced gender of users |
|  |  | Balanced age distribution of users |
|  |  | Widely distribution of users |
|  |  | Balanced classification of the user's city |
|  | Communication recognition | Likes volume |
|  |  | Collection volume |
|  | Communication participation | Comments volume |
|  |  | Repost volume |
|  | Social attention | Become a hot search recommended by the platform |
|  |  | Reposted by official media or professional health science media |
|  |  | The comments or comments are positive |

# Appendix S6. The level of agreement and modification of indicators in the round 1 NGT.

| Item | Evaluation indicators | Disagreement | Neutral | Agreement | The details information from the NGT |
| --- | --- | --- | --- | --- | --- |
| 1 | Communicator | 0.00% | 0.00% | 100.00% |  |
| 2 | Communication content | 0.00% | 0.00% | 100.00% |  |
| 3 | Communication channel | 9.09% | 9.09% | 81.82% |  |
| 4 | Communication target | 9.09% | 27.27% | 63.64% |  |
| 5 | Communication effect | 0.00% | 18.18% | 81.82% |  |
| 1.1 | Reliability | 0.00% | 9.09% | 90.91% | Being revised into “credibility” |
| 1.2 | Influence | 18.18% | 18.18% | 63.64% |  |
| 1.3 | Authoritative | 0.00% | 9.09% | 90.91% |  |
| 2.1 | Scientific | 0.00% | 0.00% | 100.00% |  |
| 2.2 | Reliability | 0.00% | 9.09% | 90.91% |  |
| 2.3 | Attractiveness | 0.00% | 9.09% | 90.91% |  |
| 2.4 | Watchability | 0.00% | 0.00% | 100.00% |  |
| 2.5 | Understandability | 9.09% | 0.00% | 90.91% |  |
| 2.6 | Actionability | 0.00% | 18.18% | 81.82% |  |
| 3.1 | Availability | 0.00% | 9.09% | 90.91% |  |
| 3.2 | Interactivity | 18.18% | 0.00% | 81.82% |  |
| 3.3 | Influence | 9.09% | 27.27% | 63.64% |  |
| 3.4 | Authoritative | 9.09% | 27.27% | 63.64% |  |
| 4.1 | Perceived cultural appropriateness | 9.09% | 36.36% | 54.55% |  |
| 4.2 | Perceived usefulness | 0.00% | 18.18% | 81.82% |  |
| 4.3 | Perceived ease of use | 9.09% | 27.27% | 63.64% |  |
| 5.1 | Spread | 9.09% | 18.18% | 72.73% |  |
| 5.2 | Communication recognition | 0.00% | 18.18% | 81.82% |  |
| 5.3 | Communication participation | 9.09% | 18.18% | 72.73% |  |
| 5.4 | Social attention | 0.00% | 9.09% | 90.91% |  |
| 1.1.1 | Account basic information disclosure | 0.00% | 0.00% | 100.00% |  |
| 1.1.2 | Account credibility | 9.09% | 0.00% | 90.91% |  |
| 1.1.3 | Whether the account's motivation for posting is health knowledge | 18.18% | 18.18% | 63.64% |  |
| 1.1.4 | Whether the account holder is engaged in medical and health-related industries | 0.00% | 18.18% | 81.82% |  |
| 1.2.1 | Account's total number of followers | 9.09% | 36.36% | 54.55% |  |
| 1.2.2 | Account's total number of received likes | 9.09% | 18.18% | 72.73% |  |
| 1.2.3 | Account's total number of posted videos | 9.09% | 36.36% | 54.55% |  |
| 1.3.1 | Account type's authority | 18.18% | 18.18% | 63.64% |  |
| 1.3.2 | Account's authentication credentials | 9.09% | 27.27% | 63.64% |  |
| 1.3.3 | Account's subject-related expertise | 0.00% | 27.27% | 72.73% |  |
| 2.1.1 | Content's consistency with modern medical consensus and scientific common sense | 0.00% | 0.00% | 100.00% |  |
| 2.1.2 | Content's political orientation, value orientation and complies with ethical standards | 0.00% | 0.00% | 100.00% |  |
| 2.1.3 | Attention to privacy | 0.00% | 27.27% | 72.73% |  |
| 2.1.4 | Unbiased information | 0.00% | 27.27% | 72.73% |  |
| 2.1.5 | The content is objective facts, not personal opinions | 9.09% | 18.18% | 72.73% |  |
| 2.1.6 | The content has a certain degree of originality | 0.00% | 27.27% | 72.73% |  |
| 2.2.1 | The content's creator's information is fully public | 0.00% | 27.27% | 72.73% |  |
| 2.2.2 | Reference information source is open | 9.09% | 0.00% | 90.91% |  |
| 2.2.3 | No ads or potentially harmful information | 0.00% | 27.27% | 72.73% |  |
| 2.2.4 | Content has info that’s kind of needed and related stuff | 9.09% | 9.09% | 81.82% |  |
| 2.2.5 | Content contains supported evidence | 0.00% | 27.27% | 72.73% |  |
| 2.3.1 | Attractive video description | 0.00% | 27.27% | 72.73% |  |
| 2.3.2 | Attractive video cover design | 9.09% | 18.18% | 72.73% |  |
| 2.3.3 | Content is in line with public health concerns | 0.00% | 0.00% | 100.00% |  |
| 2.3.4 | Content focuses on hot topics that were popular when the video was released | 0.00% | 18.18% | 81.82% |  |
| 2.3.5 | Content design is both entertaining and educational, and interesting | 0.00% | 27.27% | 72.73% |  |
| 2.4.1 | Proper use of images, text and other materials | 0.00% | 0.00% | 100.00% |  |
| 2.4.2 | Suitable duration | 18.18% | 9.09% | 72.73% |  |
| 2.4.3 | Clear image quality | 9.09% | 0.00% | 90.91% |  |
| 2.4.4 | Vivid and beautiful colors | 9.09% | 18.18% | 72.73% |  |
| 2.4.5 | Clear dubbing, with the louder volume than the background music | 9.09% | 9.09% | 81.82% |  |
| 2.4.6 | Smooth dubbing, without any lag or ambiguity | 0.00% | 9.09% | 90.91% |  |
| 2.4.7 | Appropriate use of music and sound effects | 18.18% | 9.09% | 72.73% |  |
| 2.4.8 | Suitable shooting and post-production techniques | 18.18% | 9.09% | 72.73% |  |
| 2.4.9 | Rich creative forms | 9.09% | 18.18% | 72.73% |  |
| 2.4.10 | Clear subtitles | 9.09% | 18.18% | 72.73% |  |
| 2.5.1 | Clear theme | 9.09% | 0.00% | 90.91% |  |
| 2.5.2 | Have a title that matches the subject | 9.09% | 18.18% | 72.73% |  |
| 2.5.3 | The title should have an appropriate number of words | 18.18% | 18.18% | 63.64% |  |
| 2.5.4 | The cover is consistent with the subject content | 18.18% | 9.09% | 72.73% |  |
| 2.5.5 | Have clear target users | 9.09% | 18.18% | 72.73% |  |
| 2.5.6 | Concise and not redundant information | 18.18% | 9.09% | 72.73% |  |
| 2.5.7 | Have informative subtitles | 9.09% | 27.27% | 63.64% |  |
| 2.5.8 | Videos longer than 3 minutes have a clear summary at the end | 18.18% | 18.18% | 63.64% |  |
| 2.5.9 | The number of core information is 3 to 5 | 18.18% | 27.27% | 54.55% |  |
| 2.5.10 | Core information highlights | 0.00% | 27.27% | 72.73% |  |
| 2.5.11 | Use visual cues for key information | 0.00% | 27.27% | 72.73% |  |
| 2.5.12 | The language is clear and consistent | 0.00% | 0.00% | 100.00% |  |
| 2.5.13 | The characters in the video are consistent | 9.09% | 18.18% | 72.73% |  |
| 2.5.14 | Consistent dubbing and the content of the picture | 18.18% | 9.09% | 72.73% |  |
| 2.5.15 | Consistent dubbing and subtitles | 18.18% | 9.09% | 72.73% |  |
| 2.5.16 | Expression uses unclear or confusing language | 18.18% | 9.09% | 72.73% |  |
| 2.5.17 | Understandable language | 0.00% | 0.00% | 100.00% |  |
| 2.5.18 | Clear explanation for the meaning of numbers, units and mathematical operations | 18.18% | 36.36% | 45.45% |  |
| 2.5.19 | Photos, images, tables or models to support popular science content | 0.00% | 27.27% | 72.73% |  |
| 2.6.1 | Provide practical medical advice, behavioural guidance or operation technology | 18.18% | 9.09% | 72.73% |  |
| 2.6.2 | Direct medical advice, behavioural guidance, or operational techniques to the target audience | 9.09% | 18.18% | 72.73% |  |
| 2.6.3 | Present the popular science content of behavioural guidance or operation techniques in a logical order with clear steps | 0.00% | 0.00% | 100.00% |  |
| 2.6.4 | Clearly explain how to take action using photos, graphics, tables or models | 0.00% | 9.09% | 90.91% |  |
| 3.1.1 | Reasonable and clear navigation structure | 9.09% | 18.18% | 72.73% | Reasonable navigation structure |
| 3.1.2 | Search results comprehensiveness | 9.09% | 27.27% | 63.64% |  |
| 3.1.3 | Search results accuracy | 0.00% | 27.27% | 72.73% |  |
| 3.1.4 | Stable and effective video information source | 0.00% | 9.09% | 90.91% |  |
| 3.2.1 | Available and easy to use forwarding function | 0.00% | 0.00% | 100.00% | The like and forwarding function both refer to the approval of the video, which can be merged into one indicator. |
| 3.2.2 | Available and easy to use like function | 0.00% | 9.09% | 90.91% |  |
| 3.2.3 | Available and easy to use comments and messages | 9.09% | 9.09% | 81.82% | The comments and messaging function both refer to the participation of the video, which can be merged into one indicator. |
| 3.2.4 | Available and easy to use messaging function | 0.00% | 0.00% | 100.00% |  |
| 3.3.1 | Number of platform users | 0.00% | 27.27% | 72.73% |  |
| 3.3.2 | Frequency of platform being forwarded by mainstream media | 9.09% | 18.18% | 72.73% |  |
| 3.3.3 | The proportion of health popularization videos on the platform | 0.00% | 27.27% | 72.73% |  |
| 3.4.1 | The platform has a clear and strict video review mechanism | 0.00% | 27.27% | 72.73% |  |
| 3.4.2 | Number of health certification accounts on the platform | 9.09% | 27.27% | 63.64% |  |
| 4.1.1 | Video respects your custom background | 0.00% | 27.27% | 72.73% |  |
| 4.1.2 | Video respects your cultural traditions | 9.09% | 27.27% | 63.64% |  |
| 4.1.3 | Video respects your language habits | 9.09% | 27.27% | 63.64% |  |
| 4.2.1 | Video provides you with health science knowledge | 0.00% | 0.00% | 100.00% |  |
| 4.2.2 | Video meets your health information needs | 9.09% | 18.18% | 72.73% |  |
| 4.2.3 | Video makes you feel the need to take action | 0.00% | 0.00% | 100.00% |  |
| 4.2.4 | You will change your behavior based on this video | 9.09% | 9.09% | 81.82% |  |
| 4.2.5 | You'd like to recommend this video to others | 0.00% | 27.27% | 72.73% |  |
| 4.3.1 | You can get health information in the video | 9.09% | 18.18% | 72.73% |  |
| 4.3.2 | You can understand the health information in the video | 9.09% | 18.18% | 72.73% |  |
| 4.3.3 | You can apply the health information in the video | 0.00% | 27.27% | 72.73% |  |
| 5.1.1 | Balanced gender of users | 9.09% | 36.36% | 54.55% |  |
| 5.1.2 | Balanced age distribution of users | 9.09% | 36.36% | 54.55% |  |
| 5.1.3 | Widely distribution of users | 0.00% | 36.36% | 63.64% |  |
| 5.1.4 | Balanced classification of the user's city | 9.09% | 36.36% | 54.55% |  |
| 5.2.1 | Likes volume | 0.00% | 0.00% | 100.00% | The number of views is an important indicator of video effect and needs to be added. |
| 5.2.2 | Collection volume | 0.00% | 0.00% | 100.00% |  |
| 5.3.1 | Comments volume | 9.09% | 9.09% | 81.82% |  |
| 5.3.2 | Repost volume | 9.09% | 18.18% | 72.73% |  |
| 5.4.1 | Become a hot search recommended by the platform | 0.00% | 0.00% | 100.00% |  |
| 5.4.2 | Reposted by official media or professional health science media | 0.00% | 0.00% | 100.00% |  |
| 5.4.3 | The comments or comments are positive | 9.09% | 18.18% | 72.73% | The positive ratio of comments is more actionable when making evaluations. |

# Appendix S7. The level of agreement and modification of indicators in the round 2 NGT.

| Item | Evaluation indicators | Disagreement | Neutral | Agreement |
| --- | --- | --- | --- | --- |
| 4 | Communication target | 82% | 18% | 0% |
| 1.2 | Influence | 82% | 18% | 0% |
| 3.3 | Influence | 91% | 9% | 9% |
| 3.4 | Authoritative | 100% | 0% | 0% |
| 4.1 | Perceived cultural appropriateness | 91% | 9% | 0% |
| 4.3 | Perceived ease of use | 100% | 0% | 0% |
| 5.1 | Spread | 91% | 9% | 0% |
| 5.3 | Communication participation | 91% | 0% | 9% |
| 1.1.3 | Whether the account's motivation for posting is health knowledge | 91% | 9% | 0% |
| 1.2.1 | Account's total number of followers | 100% | 0% | 0% |
| 1.2.2 | Account's total number of received likes | 100% | 0% | 0% |
| 1.2.3 | Account's total number of posted videos | 100% | 0% | 0% |
| 1.3.1 | Account type's authority | 100% | 0% | 0% |
| 1.3.2 | Account's authentication credentials | 0% | 18% | 82% |
| 1.3.3 | Account's subject-related expertise | 0% | 9% | 91% |
| 2.1.3 | Attention to privacy | 91% | 9% | 0% |
| 2.1.4 | Unbiased information | 91% | 0% | 9% |
| 2.1.5 | The content is objective facts, not personal opinions | 91% | 0% | 9% |
| 2.1.6 | The content has a certain degree of originality | 91% | 0% | 9% |
| 2.2.1 | The content's creator's information is fully public | 100% | 0% | 0% |
| 2.2.3 | No ads or potentially harmful information | 82% | 9% | 0% |
| 2.2.5 | Content contains supported evidence | 91% | 0% | 9% |
| 2.3.1 | Attractive video description | 82% | 18% | 0% |
| 2.3.2 | Attractive video cover design | 91% | 0% | 9% |
| 2.3.5 | Content design is both entertaining and educational, and interesting | 91% | 9% | 0% |
| 2.4.2 | Suitable duration | 91% | 9% | 0% |
| 2.4.4 | Vivid and beautiful colors | 91% | 9% | 0% |
| 2.4.7 | Appropriate use of music and sound effects | 91% | 9% | 0% |
| 2.4.8 | Suitable shooting and post-production techniques | 91% | 0% | 9% |
| 2.4.9 | Rich creative forms | 91% | 0% | 9% |
| 2.4.10 | Clear subtitles | 82% | 9% | 9% |
| 2.5.2 | Have a title that matches the subject | 82% | 9% | 9% |
| 2.5.3 | The title should have an appropriate number of words | 82% | 18% | 0% |
| 2.5.4 | The cover is consistent with the subject content | 82% | 18% | 0% |
| 2.5.5 | Have clear target users | 100% | 0% | 0% |
| 2.5.6 | Concise and not redundant information | 100% | 0% | 0% |
| 2.5.7 | Have informative subtitles | 91% | 9% | 0% |
| 2.5.8 | Videos longer than 3 minutes have a clear summary at the end | 100% | 0% | 0% |
| 2.5.9 | The number of core information is 3 to 5 | 91% | 9% | 0% |
| 2.5.10 | Core information highlights | 91% | 9% | 0% |
| 2.5.11 | Use visual cues for key information | 91% | 9% | 0% |
| 2.5.13 | The characters in the video are consistent | 91% | 9% | 0% |
| 2.5.14 | Consistent dubbing and the content of the picture | 82% | 9% | 9% |
| 2.5.15 | Consistent dubbing and subtitles | 82% | 9% | 9% |
| 2.5.16 | Expression uses unclear or confusing language | 82% | 9% | 9% |
| 2.5.18 | Clear explanation for the meaning of numbers, units and mathematical operations | 91% | 0% | 9% |
| 2.5.19 | Photos, images, tables or models to support popular science content | 91% | 0% | 9% |
| 2.6.1 | Provide practical medical advice, behavioural guidance or operation technology | 100% | 0% | 0% |
| 2.6.2 | Direct medical advice, behavioural guidance, or operational techniques to the target audience | 91% | 9% | 0% |
| 3.1.1 | Reasonable and clear navigation structure | 9% | 9% | 82% |
| 3.1.2 | Search results comprehensiveness | 91% | 9% | 0% |
| 3.1.3 | Search results accuracy | 91% | 0% | 9% |
| 3.2.1 | Available and easy to use like and forwarding function | 100% | 0% | 0% |
| 3.2.3 | Available and easy to use comments and messages function | 91% | 9% | 0% |
| 3.3.1 | Number of platform users | 9% | 9% | 82% |
| 3.3.2 | Frequency of platform being forwarded by mainstream media | 82% | 9% | 9% |
| 3.3.3 | The proportion of health popularization videos on the platform | 91% | 0% | 9% |
| 3.4.1 | The platform has a clear and strict video review mechanism | 91% | 0% | 9% |
| 3.4.2 | Number of health certification accounts on the platform | 91% | 0% | 9% |
| 4.1.1 | Video respects your custom background | 100% | 0% | 0% |
| 4.1.2 | Video respects your cultural traditions | 82% | 9% | 9% |
| 4.1.3 | Video respects your language habits | 91% | 9% | 0% |
| 4.2.2 | Video meets your health information needs | 91% | 0% | 9% |
| 4.2.5 | You'd like to recommend this video to others | 18% | 0% | 82% |
| 4.3.1 | You can get health information in the video | 91% | 0% | 9% |
| 4.3.2 | You can understand the health information in the video | 100% | 0% | 0% |
| 4.3.3 | You can apply the health information in the video | 100% | 0% | 0% |
| 5.1.1 | Balanced gender of users | 82% | 9% | 9% |
| 5.1.2 | Balanced age distribution of users | 82% | 9% | 9% |
| 5.1.3 | Widely distribution of users | 82% | 0% | 18% |
| 5.1.4 | Balanced classification of the user's city | 91% | 9% | 0% |
|  | Views volume | 91% | 9% | 0% |
| 5.3.1 | Comments volume | 100% | 0% | 0% |
| 5.4.2 | Reposted by official media or professional health science media | 100% | 0% | 0% |
| 5.4.3 | The positive ratio of comments | 82% | 9% | 9% |

# **Appendix S8.** Weight analysis of the tertiary indicators.

| Primary, secondary, and tertiary indicators | Weight | Rank | Overall weight | Overall rank |
| --- | --- | --- | --- | --- |
| (Who) Communicator |  |  |  |  |
| Credibility |  |  |  |  |
| Account’s basic information is disclosed | 0.19 | 3 | 0.01 | —^a^ |
| Account’s credibility is high | 0.31 | 2 | 0.02 | — |
| Account’s holder is engaged in medical and health-related domain | 0.50 | 1 | 0.03 | — |
| Authoritative |  |  |  |  |
| Account’s platform certification is authoritative | 0.34 | 2 | 0.01 | — |
| Account’s holder is expertise with specialized academic background | 0.66 | 1 | 0.03 | — |
| (What) Communication content |  |  |  |  |
| Sciencificity |  |  |  |  |
| Content’s consistency with scientific common sense | 0.50 | 1 | 0.06 | 3 |
| Content’s complies with ethical standards | 0.50 | 1 | 0.06 | 3 |
| Reliability |  |  |  |  |
| Content contains visible reference information | 0.37 | 2 | 0.03 | — |
| Content has info that’s kind of needed and related stuff | 0.63 | 1 | 0.05 | 5 |
| Attractiveness |  |  |  |  |
| Content aligns with people’s health concerns | 0.55 | 1 | 0.03 | — |
| Content focuses on popular health topics | 0.45 | 2 | 0.02 | — |
| Watchability |  |  |  |  |
| Images, text and other materials are used properly | 0.32 | 1 | 0.02 | — |
| Image quality is clear | 0.21 | 3 | 0.01 | — |
| Dubbing is clear, louder than background music | 0.18 | 4 | 0.01 | — |
| Dubbing is smooth, without any lag or ambiguity | 0.28 | 2 | 0.02 | — |
| Understandability |  |  |  |  |
| Theme is clear | 0.37 | 2 | 0.03 | 10 |
| Expression is clear and consistent | 0.20 | 3 | 0.02 | — |
| Expression uses unclear or confusing language | 0.43 | 1 | 0.04 | 9 |
| Actionability |  |  |  |  |
| Behavioral guidance or operation techniques are presented with clear steps in logical order | 0.56 | 1 | 0.04 | 8 |
| Photos, graphics, tables or models are used to explain how to take action clearly | 0.44 | 2 | 0.03 | — |
| (In which channel) Communication channel |  |  |  |  |
| Availability |  |  |  |  |
| Reasonable and clear navigation structure | 0.32 | 2 | 0.02 | — |
| Stable and effective video information source | 0.68 | 1 | 0.05 | 6 |
| Interactivity |  |  |  |  |
| Available and easy to use like and share functions | 0.60 | 2 | 0.02 | — |
| Available and easy to use comments and messages functions | 0.40 | 1 | 0.01 | — |
| (With what effect) Communication effect |  |  |  |  |
| Perceived usefulness |  |  |  |  |
| Perception of knowledge acquisition | 0.25 | 2 | 0.03 | — |
| Perception of action necessity | 0.21 | 4 | 0.03 | — |
| Intention of behavioral modification | 0.24 | 3 | 0.03 | — |
| Motivation to share | 0.30 | 1 | 0.04 | 7 |
| Engagement |  |  |  |  |
| Views volume | 0.36 | 1 | 0.03 | — |
| Likes volume | 0.19 | 4 | 0.02 | — |
| Collection volume | 0.21 | 3 | 0.02 | — |
| Repost volume | 0.24 | 2 | 0.02 | — |
| Social attention |  |  |  |  |
| Recommended as a trending video | 0.53 | 1 | 0.07 | 1 |
| Positive comments ratio | 0.47 | 2 | 0.07 | 2 |

# Appendix S9. Analysis of the matrix and weight of the secondary indicator.

| Primary indicators | Secondary indicators |  |  |  |  |  |  | Weight | Rank | Combined weight | CI |
| --- | --- | --- | --- | --- | --- | --- | --- | --- | --- | --- | --- |
| (Who) Communicator |  | Credibility | Authoritative |  |  |  |  |  |  |  | 0 |
|  | Credibility | 1 | 1.3077 |  |  |  |  | 0.5667 | 1 | 0.0556 |  |
|  | Authoritative | 0.7647 | 1 |  |  |  |  | 0.4333 | 2 | 0.0425 |  |
| (What) Communication Content |  | Scientific | Reliability | Attractiveness | Watchability | Understandability | Actionability |  |  |  | 0.0904 |
|  | Scientific | 1 | 1.3473 | 2.1037 | 1.8678 | 1.3051 | 1.6571 | 0.2426 | 1 | 0.1108 |  |
|  | Reliability | 0.7422 | 1 | 1.5615 | 1.3864 | 0.9687 | 1.23 | 0.18 | 3 | 0.0822 |  |
|  | Attractiveness | 0.4754 | 0.6404 | 1 | 0.8879 | 0.6204 | 0.7877 | 0.1153 | 6 | 0.0527 |  |
|  | Watchability | 0.5354 | 0.7213 | 1.1263 | 1 | 0.6988 | 0.8872 | 0.1299 | 5 | 0.0593 |  |
|  | Understandability | 0.7662 | 1.0323 | 1.6119 | 1.4311 | 1 | 1.2697 | 0.1859 | 2 | 0.0849 |  |
|  | Actionability | 0.6035 | 0.813 | 1.2695 | 1.1271 | 0.7876 | 1 | 0.1464 | 4 | 0.0669 |  |
| (In which channel) Communication Channel |  | Availability | Interactivity |  |  |  |  |  |  |  | 0 |
|  | Availability | 1 | 2.9403 |  |  |  |  | 0.7462 | 1 | 0.0741 |  |
|  | Interactivity | 0.3401 | 1 |  |  |  |  | 0.2538 | 2 | 0.0252 |  |
| (With what effect) Communication Effect |  | Perceived usefulness | Engagement | Social attention |  |  |  |  |  |  | 0.0135 |
|  | Perceived usefulness | 1 | 1.5088 | 0.9063 |  |  |  | 0.3615 | 2 | 0.125 |  |
|  | Engagement | 0.6628 | 1 | 0.6007 |  |  |  | 0.2396 | 3 | 0.0829 |  |
|  | Social attention | 1.1034 | 1.6648 | 1 |  |  |  | 0.3989 | 1 | 0.1379 |  |

# Appendix S10. Demographic characteristics of pilot study participants

| ID | Role | Age | Gender | Education Level | Notes on HESV Engagement |
| --- | --- | --- | --- | --- | --- |
| V01 | Viewer | 19 | Female | Undergraduate | Regular watches HESVs |
| V02 | Viewer | 46 | Male | Associate degree | Occasionally watches HESVs |
| V03 | Viewer | 30 | Female | Associate degree | Frequently watches HESVs |
| V04 | Viewer | 19 | Male | Undergraduate | Regular watches HESVs |
| V05 | Viewer | 20 | Female | Undergraduate | Frequently watches HESVs |
| V06 | Viewer | 20 | Female | Undergraduate | Frequently watches HESVs |
| V07 | Viewer | 30 | Female | Undergraduate | Frequently watches HESVs |
| V08 | Viewer | 20 | Female | Undergraduate | Actively follows HESVs on a daily basis |
| V09 | Viewer | 37 | Female | Associate degree | Actively follows HESVs on a daily basis |
| P10 | Producer | 33 | Male | Master’s degree | Professional content creator |
| P11 | Producer | 36 | Female | Master’s degree | Works in health communication |
| P12 | Producer | 42 | Female | Doctorate (PhD) | Academic health educator |
| P13 | Producer | 39 | Female | Master’s degree | Professional content creator |
| P14 | Producer | 54 | Male | Doctorate (MD) | Public health lecturer |
| P15 | Producer | 38 | Female | Doctorate (MD) | Editor for medical science videos |

**References**

1. 奚道佳. 移动社交媒体健康信息质量评价与治理策略研究: 山东财经大学; 2021.

2. 戴萍萍. 移动社交媒体健康信息质量评价体系研究 [博士]2022.

3. 陈浩, 李银胜. 面向多维度的电子商务主体信誉评价与计算. In: 陈浩, 李银胜, editors. 计算机应用与软件2015. p. 26-30.

4. 国佳, 郭勇, 沈旺, 潘梦雅. 基于在线评论的网络社区信息可信度评价方法研究. 图书情报工作2019. p. 137-44.

5. Wang Z, Walther JB, Pingree S, Hawkins RP. Health Information, Credibility, Homophily, and Influence via the Internet: Web Sites Versus Discussion Groups. Health Communication. 2008 2008/08/19;23(4):358-68. doi: 10.1080/10410230802229738.

6. Kong W, Song S, Zhao YC, Zhu Q, Sha L. TikTok as a Health Information Source: Assessment of the Quality of Information in Diabetes-Related Videos. J Med Internet Res. 2021 2021/9/1;23(9):e30409. doi: 10.2196/30409.

7. Sillence E, Briggs P, Harris PR, Fishwick L. How do patients evaluate and make use of online health information? Social Science & Medicine. 2007 2007/05/01/;64(9):1853-62. doi: <https://doi.org/10.1016/j.socscimed.2007.01.012>.

8. 熊敏. 健康科普视频用户体验及影响因素分析 [硕士]2021.

9. 曲云丽. 抖音健康类短视频信息可信度评价研究 [硕士]2022.

10. Neiger BL, Thackeray R, Burton SH, Giraud-Carrier CG, Fagen MC. Evaluating social media's capacity to develop engaged audiences in health promotion settings: use of Twitter metrics as a case study. Health Promot Pract. 2013 Mar;14(2):157-62. PMID: 23271716. doi: 10.1177/1524839912469378.

11. 严小芳. 移动短视频的传播特性和媒体机遇. 东南传播. 2016 (02):90-2. doi: 10.13556/j.cnki.dncb.cn35-1274/j.2016.02.032.

12. 王秀丽, 赵雯雯, 袁天添. 社会化媒体效果测量与评估指标研究综述. In: 王秀丽, 赵雯雯, 袁天添, editors. 国际新闻界2017. p. 6-24.

13. 陈海波, 杨晓雯, 陈万超. 基于系统动力学的图书馆网络健康信息有效传播形成机理研究. 四川图书馆学报2024. p. 48-54.

14. 柯婷娟, 曾桢. 不同主题下农村信息传播存在问题和影响因素综述. In: 柯婷娟, 曾桢, editors. 农业图书情报学报2022. p. 14-26.

15. Wathen CN, Burkell J. Believe it or not: Factors influencing credibility on the Web. Journal of the American Society for Information Science and Technology. 2002;53(2):134-44. doi: <https://doi.org/10.1002/asi.10016>.

16. 郝玉佩. 短视频中的健康传播探讨——以“丁香医生”抖音号为例. 新闻世界. 2019 (02):75-7. doi: 10.19497/j.cnki.1005-5932.2019.02.022.

17. Lucassen T, Schraagen JM. Factual accuracy and trust in information: The role of expertise. Journal of the American Society for Information Science and Technology. 2011;62(7):1232-42. doi: <https://doi.org/10.1002/asi.21545>.

18. Rieh SY. Judgment of information quality and cognitive authority in the Web. Journal of the American Society for Information Science and Technology. 2002;53(2):145-61. doi: <https://doi.org/10.1002/asi.10017>.

19. Azer SA. Are DISCERN and JAMA Suitable Instruments for Assessing YouTube Videos on Thyroid Cancer? Methodological Concerns. Journal of Cancer Education. 2020 2020/12/01;35(6):1267-77. doi: 10.1007/s13187-020-01763-9.

20. 聂静虹. 健康传播学. 聂静虹, editor. 广州: 中山大学出版社; 2019. ISBN: 9787306065926;7306065920;.

21. 邢子鑫. Bilibili平台健康板块视频传播效果影响因素研究 [硕士]2021.

22. 李长宁. 健康传播材料制作与评价. 李长宁, editor. 北京: 人民卫生出版社; 2018. ISBN: 7117266449;9787117266444;.

23. Egala SB, Liang D, Boateng D. Social Media Health-Related Information Credibility and Reliability: An Integrated User Perceived Quality Assessment. IEEE Transactions on Engineering Management. 2024;71:5018-29. doi: 10.1109/TEM.2022.3225182.

24. 国卫宣传发〔2022〕11号. 关于建立健全全媒体健康科普知识发布和传播机制的指导意见. 2022; Available from: <http://www.nhc.gov.cn/xcs/s3581/202205/1c67c12c86b44fd2afb8e424a2477091.shtml>.

25. 严梦. 健康类微信公众号信息质量评价指标体系研究 [硕士]2020.

26. Kocyigit BF, Akaltun MS. Does YouTube provide high quality information? Assessment of secukinumab videos. Rheumatology International. 2019 2019/07/01;39(7):1263-8. doi: 10.1007/s00296-019-04322-8.

27. 宣传司. 健康科普信息生成与传播技术指南（试行）. 2015 [updated 2015-8-11; cited 2024 Apr. 14]; Available from: <http://www.nhc.gov.cn/xcs/s3581/201508/5fe32b5a1a8243e2bd819f9eeebfd8b1.shtml>.

28. 李明, 李莹, 许应成. 突发事件环境下的虚拟问答社区知识可信度影响因素研究. In: 李明, 李莹, 许应成, editors. 情报理论与实践2019. p. 128-32+45.

29. Winker MA, Flanagin A, Chi-Lum B, White J, Andrews K, Kennett RL, et al. Guidelines for Medical and Health Information Sites on the Internet Principles Governing AMA Web Sites. JAMA. 2000;283(12):1600-6. doi: 10.1001/jama.283.12.1600.

30. 隋雨佳. 社会化媒体中伪健康信息的治理策略研究 [博士]2021.

31. 侯震, 童惟依, 邓靖飞, 李扬. 新媒体环境下健康科普视频的创作与传播策略研究. In: 侯震, 童惟依, 邓靖飞, 李扬, editors. 中国医学教育技术2022. p. 51-4.

32. Charnock D, Shepperd S, Needham G, Gann R. DISCERN: an instrument for judging the quality of written consumer health information on treatment choices. Journal of epidemiology and community health. 1999 Feb;53(2):105-11. PMID: 10396471. doi: 10.1136/jech.53.2.105.

33. 中国科普作家网. 中国科普作家协会《科普视频评价指标体系》等两项团体标准正式发布. 2023; Available from: <https://www.kpcswa.org.cn/web/press/news/102AO62023.html>.

34. Chai BS, Ingledew P-A. Assessment of Lung Cancer YouTube Videos for Patient Education. Journal of Cancer Education. 2023 2023/10/01;38(5):1760-6. doi: 10.1007/s13187-023-02332-6.

35. Dutta-Bergman MJ. The Impact of Completeness and Web Use Motivation on the Credibility of e-Health Information. Journal of Communication. 2004 2004/06/01;54(2):253-69. doi: <https://doi.org/10.1111/j.1460-2466.2004.tb02627.x>.

36. Lederman R, Fan H, Smith S, Chang S. Who can you trust? Credibility assessment in online health forums. Health Policy and Technology. 2014 2014/03/01/;3(1):13-25. doi: <https://doi.org/10.1016/j.hlpt.2013.11.003>.

37. 周海赟, 张舒. 公安宣传类短视频封面质量提升策略研究. In: 周海赟, 张舒, editors. 新闻世界2024. p. 17-20.

38. 李怡青. 健康传播类短视频的叙事研究 [硕士]2023.

39. Xu J, Benbasat I, Cenfetelli RT. Integrating Service Quality with System and Information Quality: An Empirical Test in the E-Service Context. MIS Q. 2013;37:777-94.

40. 王梦瑶. 科普微视频评价标准研究 [硕士]2018.

41. 张冉. B站健康科普类短视频传播效果影响因素研究. 新闻世界. 2023 (05):11-5. doi: 10.19497/j.cnki.1005-5932.2023.05.017.

42. 黄宇. 抖音健康传播类短视频发展策略探究 [硕士]2022.

43. 冀榕. 抖音平台健康科普短视频传播效果研究 [硕士]2021.

44. Song S, Park KM, Phong K, Kim EA. Evaluating the Quality and Reliability of Gender-affirming Surgery Videos on YouTube and TikTok. Plastic and Reconstructive Surgery – Global Open. 2022;10(7):e4443. PMID: 01720096-202207000-00042. doi: 10.1097/gox.0000000000004443.

45. Shoemaker SJ, Wolf MS, Brach C. Development of the Patient Education Materials Assessment Tool (PEMAT): a new measure of understandability and actionability for print and audiovisual patient information. Patient Educ Couns. 2014 Sep;96(3):395-403. PMID: 24973195. doi: 10.1016/j.pec.2014.05.027.

46. Prevention CfDCa. CDC Clear Communication Index: A Tool for Developing and Assessing CDC Public Communication Products. 2019; Available from: <https://www.cdc.gov/ccindex/index.html>.

47. Moon H, Lee GH. Evaluation of Korean-Language COVID-19–Related Medical Information on YouTube: Cross-Sectional Infodemiology Study. J Med Internet Res. 2020 2020/8/12;22(8):e20775. doi: 10.2196/20775.

48. 闫相儒. 健康类短视频封面及标题设计对播放量的影响研究 [硕士]2023.

49. Handler SJ, Eckhardt SE, Takashima Y, Jackson AM, Truong C, Yazdany T. Readability and quality of Wikipedia articles on pelvic floor disorders. International Urogynecology Journal. 2021 2021/12/01;32(12):3249-58. doi: 10.1007/s00192-021-04776-0.

50. Communication OotADf. How to Develop Products for Adults with Intellectual Developmental Disabilities and Extreme Low Literacy: A Product Development Tool. 2023; Available from: <https://www.cdc.gov/ccindex/pdf/idd-ell-product-development-tool-508.pdf>.

51. 胡文茜. 超级IP视角下短视频内容质量评价研究 [硕士]2022.

52. Rockville. PEMAT Tool for Audiovisual Materials (PEMAT-A/V). Agency for Healthcare Research and Quality; 2020; Available from: <https://www.ahrq.gov/health-literacy/patient-education/pemat-av.html>.

53. 刘辉. 公众健康信息学. 刘辉, editor. 北京: 中国协和医科大学出版社; 2021. ISBN: 9787567917491;7567917491;.

54. Shan Y, Xing Z, Dong Z, Ji M, Wang D, Cao X. Translating and Adapting the DISCERN Instrument Into a Simplified Chinese Version and Validating Its Reliability: Development and Usability Study. J Med Internet Res. 2023 Feb 2;25:e40733. PMID: 36729573. doi: 10.2196/40733.

55. 国家统计局. 国家统计质量保证框架. 2013; Available from: <https://www.gov.cn/gzdt/2013-09/24/content_2493720.htm>.

56. 魏萌萌. 糖尿病网络健康信息的质量评估指标体系构建与实证研究 [硕士]2012.

57. Egger FN. "Trust me, I'm an online vendor": towards a model of trust for e-commerce system design. In: Egger FN, editor. CHI '00 Extended Abstracts on Human Factors in Computing Systems; The Hague, The Netherlands: Association for Computing Machinery; 2000. p. 101–2.

58. 南京大学新闻传播学院. 中国媒体融合传播效果指数. 2018; Available from: <https://jc.nju.edu.cn/99/d2/c8625a235986/page.htm>.

59. Cheng L, editor. The development of recommendations for future amniocentesis education and decision-aiding videos. 2000.

60. Klobas JE. Beyond information quality: fitness for purpose and electronic information resource use. Journal of Information Science. 1995;21(2):95-114. doi: doi: 10.1177/016555159502100204.

61. 熊成敏. 中年群体对健康科普类短视频的信息采纳意愿研究 [硕士]2022.

62. Davis FD. Perceived Usefulness, Perceived Ease of Use, and User Acceptance of Information Technology. MIS Q. 1989;13:319-40.

63. 李欣蕊. 公众HPV疫苗认知水平影响因素研究 [硕士]2021.

64. 姜萌. F民营医院公众号内容转发意愿研究 [硕士]2020.

65. 朱益平, 杜海娇, 张佳, 周赞. 基于RS-BP神经网络的政务微信公众号信息质量评价模型研究. 情报科学. 2021;39(02):54-61+9. doi: 10.13833/j.issn.1007-7634.2021.02.007.

66. 牛如意. 微名人视频植入式广告传播效果研究 [硕士]2023.

67. 曾欢欢. 《央视农业》哔哩哔哩账号传播效果及影响因素分析 [硕士]2023.

68. Li R, Rahaman MM, Tang Z, Zhao L. Assessing Social Media Communications of Local Governments in Fast-Growing U.S. Cities. The Professional Geographer. 2021 2021/09/21;73(4):702-12. doi: 10.1080/00330124.2021.1933547.

69. 匡文波, 武晓立. 基于微信公众号的健康传播效果评价指标体系研究. In: 匡文波, 武晓立, editors. 国际新闻界2019. p. 153-76.

70. 程凯歌. B站知识类视频传播效果影响因素研究 [硕士]2022.

71. 李帆. 抖音平台健康类账号的内容生产与传播效果分析 [硕士]2022.

72. 胡媛, 韦肖莹, 王灿. 微博信息质量评价指标体系构建研究. 情报科学. 2017;35(06):44-50. doi: 10.13833/j.cnki.is.2017.06.039.
