# Supplementary material for: Developing an Evaluation System for Quality of Health Educational Short Videos on Social Media (LassVQ) Using Nominal Group Technique and Analytic Hierarchy Process: Qualitative Study
Source: J Med Internet Res. 2025 Sep 18;27:e72661. doi: 10.2196/72661 (PMC12445618; doi:10.2196/72661)
Supplement: Checklist 1 [file jmir-v27-e72661-s002.docx]

Items for reporting studies on measurement properties of Patient-Reported Outcome Measures (PROMs): General Reporting recommendations relevant for all studies on measurement properties

| **General Reporting recommendations relevant for all studies on measurement properties** | | |  |
| --- | --- | --- | --- |
| Item | Item name | Item description | Reported on page # |
| Report section: Title | | |  |
| T1 | Title | Identify the report as a study of one or more measurement properties of a specific PROM to measure a specified construct in a specified population. | 1 |
| Report section: Abstract | | |  |
| A1 | Objectives | Provide the specific objective(s) of the research, specifying (1) the name (and version, if relevant), and construct(s) of the PROM, (2) the measurement properties being evaluated, and (3) relevant study characteristics. | 2 |
| A2 | Design | Specify (details of the) study design used to evaluate the measurement properties. | 2 |
| A3 | Methods | Specify the methods for evaluating each measurement property. | 2 |
| A4 | Results | Provide the main results for all measurement properties evaluated. | 2 |
| A5 | Discussion/Conclusions | Provide a brief statement of the implications of the findings in the context of existing evidence on the PROM. | 2 |
| Report section: Introduction | | |  |
| I1 | PROM | Specify the name and, if relevant, the version, and construct(s) of the PROM. | 5 |
| I2 | Target population & context of use | Specify the target population and context of use that the PROM was designed for. | 5 |
| I3 | State of knowledge & Rationale | Provide a description of the current scientific knowledge (what is known and not known) regarding the measurement properties of the PROM. Explain why the new study is necessary. Provide citations for the original development paper(s). | 4 |
| I4 | Objectives | Provide the specific objective(s) of the research, specifying (1) the name (and version, if relevant) of the PROM, (2) the measurement properties being evaluated, and (3) relevant study sample characteristics. | 4, 5 |
| Report section: General Methods | | |  |
| GM1 | Study design | Specify (details of the) study design used to evaluate the measurement properties. | 5 |
| GM2 | Participants | Specify how the study participants were selected. Specify the inclusion and exclusion criteria | 6, 7, 10 |
| GM3 | PROM details | Provide details about the original version of the PROM as well as of the PROM version being studied, specify the conceptual framework (reflective/formative model), details on the structure (the number of items and subscales), the language, response scale, recall period, direction of scoring, and scoring algorithm of the PROM. Specify how the PROM was administered (e.g., in what setting, mode of administration (e.g. paper, electronic) what instructions were given), including the country in which it is administered | 6 |
| GM4 | Additional data collection | Describe why and how other data was collected (e.g., construct and measurement properties of the comparator instruments, characteristics of groups being compared, and rationale for choosing groups), including mode of administration (e.g., paper, electronic). | 8, 9, 10 |
| GM5 | Time points procedures | Provide all time points of all measurements. | 6, 8 |
| GM6 | Justification for sample size | Provide a rationale for the sample size for all measurement properties analyses (including subgroups). | 8 |
| GM7 | Statistical analyses | Describe the statistical analyses corresponding to all objectives (see measurement properties specific boxes). Describe the criteria for good measurement properties. Name the statistical package used and the version. | 8, 9, 10 |
| GM8 | Missing data | Describe approaches for dealing with missing data. | NA |
| GM9 | Unplanned analysis | Specify analyses that were unplanned and their rationale. | NA |
| Report section: General results | | |  |
| GR1 | Participant characteristics | Provide study participants’ characteristics, specified per subgroup if applicable. | 12, 20 |
| GR2 | Sample size | Provide the total number of participants included in the study and the sample size for each analysis. | 12, 20 |
| GR3 | Missing data | Provide amount of (proportion or count) and reasons for missing data for each analysis for the PROM, and for any analyses of other outcome measurement instruments. | NA |
| GR4 | Results | Describe the results corresponding to all objectives (see measurement properties specific boxes). | 11-20 |
| Report section: Discussion/conclusions | | |  |
| DC1 | Measurement property evidence | Provide the main findings and if each measurement property is sufficient or insufficient and why. | 20, 21 |
| DC2 | Practical relevance | Discuss the practical relevance of the findings in terms of recommendations for (not) using the PROM. | 21-24 |
| DC3 | Strengths and limitations | Discuss strengths and limitations of each study. For example, discuss if there were any potential biases in the study that could have impacted the results. | 24 |
| DC4 | Generalizability | Discuss generalizability of the results. For example, discuss whether the results could be generalized to other populations given the sample studied. | 24 |
| DC5 | Instrument changes | Discuss what modifications are needed to the existing PROM. | 25 |
| DC6 | Future research | Describe new research questions or hypotheses generated from these findings, and provide/describe the research needed to answer those questions. | 25 |
| DC7 | Conclusions | Provide the overall conclusions for the use of the PROM. | 25 |
| Report section: Other information | | |  |
| O1 | Conflict of interest | State any conflict of interest you may have related to the PROM. This may include any involvement in the development of the PROM or any commercial funding or profit. | 26 |
